# Host-induced cell wall remodeling impairs opsonophagocytosis of *Staphylococcus aureus* by neutrophils

**DOI:** 10.1128/mbio.01643-24

**Published:** 2024-07-23

**Authors:** Elizabeth V. K. Ledger, Andrew M. Edwards

**Affiliations:** 1 Centre for Bacterial Resistance Biology, Imperial College London, London, United Kingdom; Institut Pasteur, Paris, France; National Institute of Allergy and Infectious Diseases, Hamilton, Montana, USA

**Keywords:** *Staphylococcus aureus*, neutrophil, antibody, peptidoglycan, opsonophagocytosis, immune evasion

## Abstract

The bacterial pathogen *Staphylococcus aureus* responds to the host environment by increasing the thickness of its cell wall. However, the impact of cell wall thickening on susceptibility to host defenses is unclear. Using bacteria incubated in human serum, we show that host-induced increases in cell wall thickness led to a reduction in the exposure of bound antibody and complement and a corresponding reduction in phagocytosis and killing by neutrophils. The exposure of opsonins bound to protein antigens or lipoteichoic acid (LTA) was most significantly reduced, while opsonization by IgG against wall teichoic acid or peptidoglycan was largely unaffected. Partial digestion of accumulated cell wall using the enzyme lysostaphin restored opsonin exposure and promoted phagocytosis and killing. Concordantly, the antibiotic fosfomycin inhibited cell wall remodeling and maintained the full susceptibility of *S. aureus* to opsonophagocytic killing by neutrophils. These findings reveal that host-induced changes to the *S. aureus* cell wall reduce the ability of the immune system to detect and kill this pathogen through reduced exposure of protein- and LTA-bound opsonins.

**IMPORTANCE:**

Understanding how bacteria adapt to the host environment is critical in determining fundamental mechanisms of immune evasion, pathogenesis, and the identification of targets for new therapeutic approaches. Previous work demonstrated that *Staphylococcus aureus* remodels its cell envelope in response to host factors and we hypothesized that this may affect recognition by antibodies and thus killing by immune cells. As expected, incubation of *S. aureus* in human serum resulted in rapid binding of antibodies. However, as bacteria adapted to the serum, the increase in cell wall thickness resulted in a significant reduction in exposure of bound antibodies. This reduced antibody exposure, in turn, led to reduced killing by human neutrophils. Importantly, while antibodies bound to some cell surface structures became obscured, this was not the case for those bound to wall teichoic acid, which may have important implications for vaccine design.

## INTRODUCTION

*Staphylococcus aureus* frequently infects wounds caused by surgery or insertion of intravenous access devices (1, 2). These infections can result in *S. aureus* seeding into the bloodstream, leading to bacteremia and subsequent metastatic dissemination to sites including the heart, bones, and joints (1, 3–5). Despite antibiotic therapy and a potent immune response, *S. aureus* infections have a high rate of relapse and frequently become chronic or recurrent (3, 5).

Neutrophils are a key host defense against *S. aureus* infection and are recruited to the site of infection from the bloodstream (6–11). The detection of *S. aureus* by neutrophils is largely dependent on the opsonization of bacteria by bound antibody and complement, which is enabled in most people by the presence of antibodies against a range of different staphylococcal surface structures, including wall and lipoteichoic acids (WTA, LTA), peptidoglycan, capsular polysaccharide, and proteins (10–18). While the precise abundance of antibodies against each of the major surface structures varies from person to person, antibodies against each macromolecule have been demonstrated to be sufficient to trigger opsonophagocytosis (14–20).

The binding of neutrophils to opsonins on the surface of *S. aureus* occurs via dedicated receptors and triggers phagocytosis of the pathogen followed by the subsequent exposure of ingested bacteria to a raft of bactericidal products including reactive oxygen species, antimicrobial peptides, and proteases (11, 12).

To combat the threat posed by neutrophils, *S. aureus* has evolved numerous mechanisms of evading opsonic complement and antibody (12, 13, 21, 22). For example, *S. aureus* produces two immunoglobulin binding proteins, Spa and Sbi, that reduce antibody-mediated opsonization, while the production of proteins such as SCIN, Efb, and CHIPS reduces complement deposition and activation and detection by immune cells (12, 13, 21–31). As such, the bacterial cell surface is a critically important determinant in immune detection of *S. aureus* and efforts by the pathogen to evade surveillance and killing by host defenses.

The staphylococcal cell envelope is a dynamic structure that responds to host-induced stresses (32–35). Consequently, *S. aureus* has a thicker cell wall *in vivo* than when growing *in vitro* (36), a phenotype that is replicated when staphylococci are exposed to human serum or present within endothelial or osteoblast cells (37–40). In the case of serum, cell wall thickening is triggered when *S. aureus* detects the presence of the host defense antimicrobial peptide LL-37 via the GraRS two-component system (37). This results in significantly greater quantities of both peptidoglycan and WTA in the cell wall, relative to bacteria grown in laboratory culture medium (37). Importantly, the changes to the cell envelope triggered by human serum are distinct from those that occur during bacterial entry into stationary phase and are also not triggered by incubation of *S. aureus* in PBS or cell culture medium, that is, serum-induced changes are not simply due to a lack of nutrients or lack of staphylococcal replication, but represent a specific response to the host environment (37).

Host-induced changes to the cell wall are important for the ability of the pathogen to cause and sustain infection. Cell wall thickening has been shown to reduce susceptibility to antibiotics, while mutant strains lacking various cell wall synthetic enzymes are less virulent in infection models (32, 34, 35, 37, 40). However, it is unknown whether host-induced changes to the bacterial cell wall affect the detection and killing of *S. aureus* by the host immune system. To address this, we examined the impact of host-induced changes to the staphylococcal cell envelope on subsequent interactions of *S. aureus* with neutrophils. This revealed that cell wall thickening constitutes a previously unrecognized mechanism of immune evasion that functions by significantly reducing the exposure of opsonins bound to proteins and LTA, thereby reducing opsonophagocytic killing.

## RESULTS

### Host-induced changes to *S. aureus* reduce killing by neutrophils

**Fig 1:**
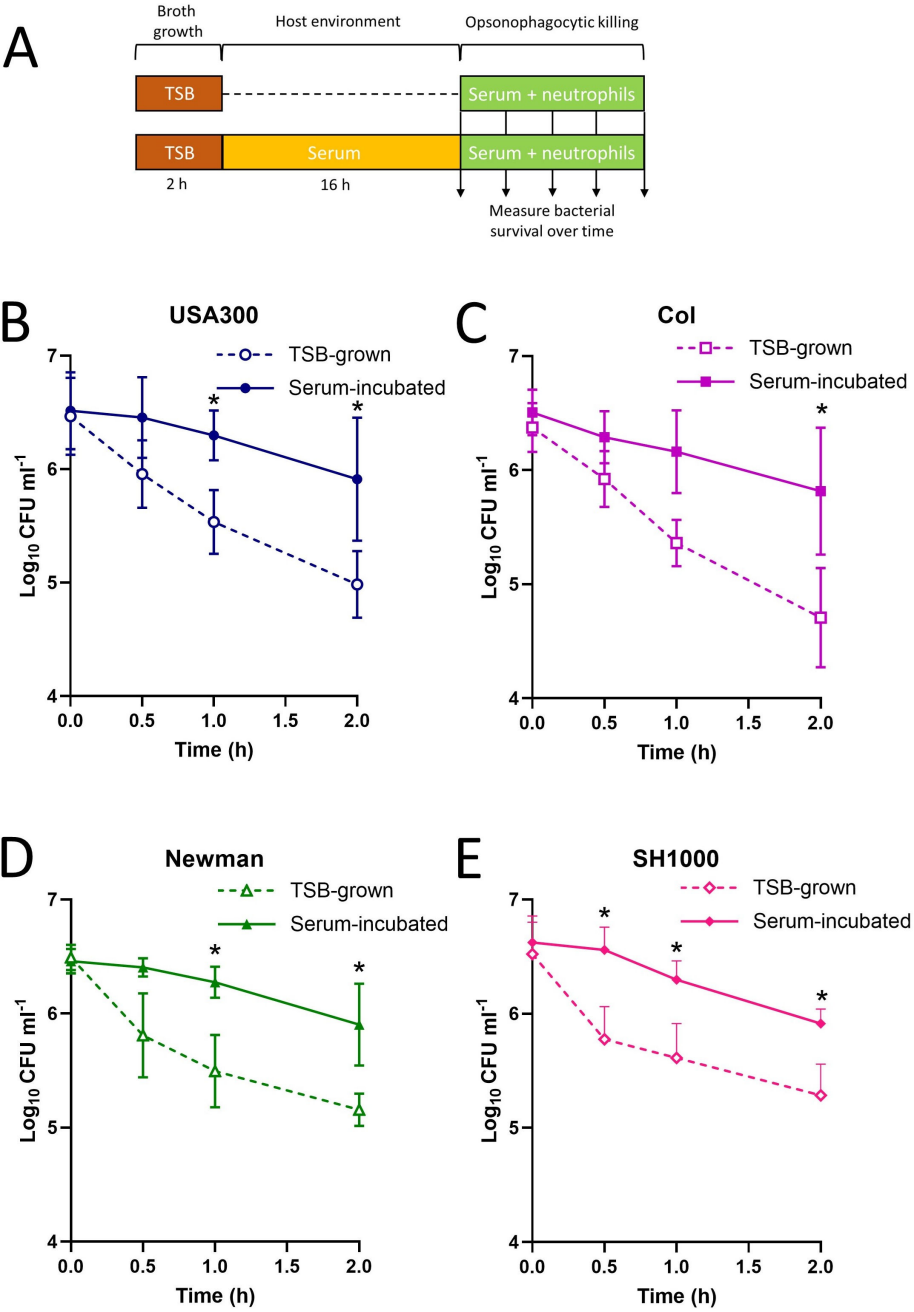
Incubation in human serum promotes tolerance to killing by neutrophils. Bacteria were grown in TSB or incubated in 100% human serum before incubation with human neutrophils in the presence of 10% human serum, and staphylococcal survival was measured over time (**A**). Survival of TSB-grown and serum-incubated cultures of *S. aureus* USA300 (**B**), Col (**C**), Newman (**D**), and SH1000 (**E**) during a 2 h incubation with purified human neutrophils. Data represent the geometric mean ± geometric standard deviation of at least three independent biological replicates. Data were analyzed by a two-way ANOVA with Sidak’s *post hoc* test (*P < 0.05; serum incubated vs TSB grown at indicated time points).

To understand the impact of the host environment on staphylococcal susceptibility to host defenses, we either grew bacteria to exponential phase in tryptic soy broth (TSB grown) to represent standard laboratory conditions or incubated *S. aureus* in 100% human serum (serum-incubated) to mimic host conditions as previously described, which triggers cell wall thickening (37) (Fig. 1A). This model uses pooled human serum, which avoids variability in anti-staphylococcal antibody levels between donors (14). Since the serum is not heat inactivated, it contains functional immunoglobulins and complement components.

In addition to triggering cell wall thickening via the GraRS system, the serum also suppresses both the growth of *S. aureus* and activation of the Agr quorum-sensing system that regulates the expression of many virulence factors (37, 41–48).

Following TSB growth or serum incubation, we then measured the survival of bacteria prepared under each condition during incubation with purified *ex vivo* human neutrophils from male and female healthy donors in the presence of fresh serum (10%) to provide antibody- and complement-mediated opsonization (9, 49). We examined four distinct wild-type *S. aureus* strains to represent both methicillin-resistant (USA300, Col) and methicillin-susceptible (SH1000, Newman) organisms (50–53).

For all four of the *S. aureus* strains tested, exponential phase bacteria were efficiently killed over time, with <5% of bacteria remaining viable after 2 h incubation with neutrophils (Fig. 1B through E). However, serum-incubated bacteria survived at levels up to five times greater than that seen for exponential bacteria for all strains (Fig. 1B through E). In addition to demonstrating that serum incubation reduced staphylococcal susceptibility to host immune defenses, the high level of consistency observed across all four strains indicated that this is a conserved phenotype.

To understand whether the bacterial growth phase was important for the reduced susceptibility of serum-incubated bacteria to neutrophil-mediated killing, we repeated the assay using USA300 grown to stationary phase. We found that serum-incubated stationary phase cells were significantly less susceptible to neutrophil-mediated killing compared to TSB-grown stationary phase *S. aureus*, with similar levels of survival to exponential phase bacteria (Fig. 1B; Fig. S1). Therefore, the protective effect of serum incubation on *S. aureus* survival during exposure to neutrophils was not dependent on the bacterial growth phase.

### Host-induced changes to *S. aureus* reduce opsonin exposure and opsonophagocytosis

Having found that serum incubation reduced staphylococcal susceptibility to host defenses relative to TSB-grown bacteria, we next determined the mechanism(s) responsible. Given the consistency in survival data across all four strains examined, we focused on the USA300 lineage since it is both well characterized and clinically important (50).

**Fig 2:**
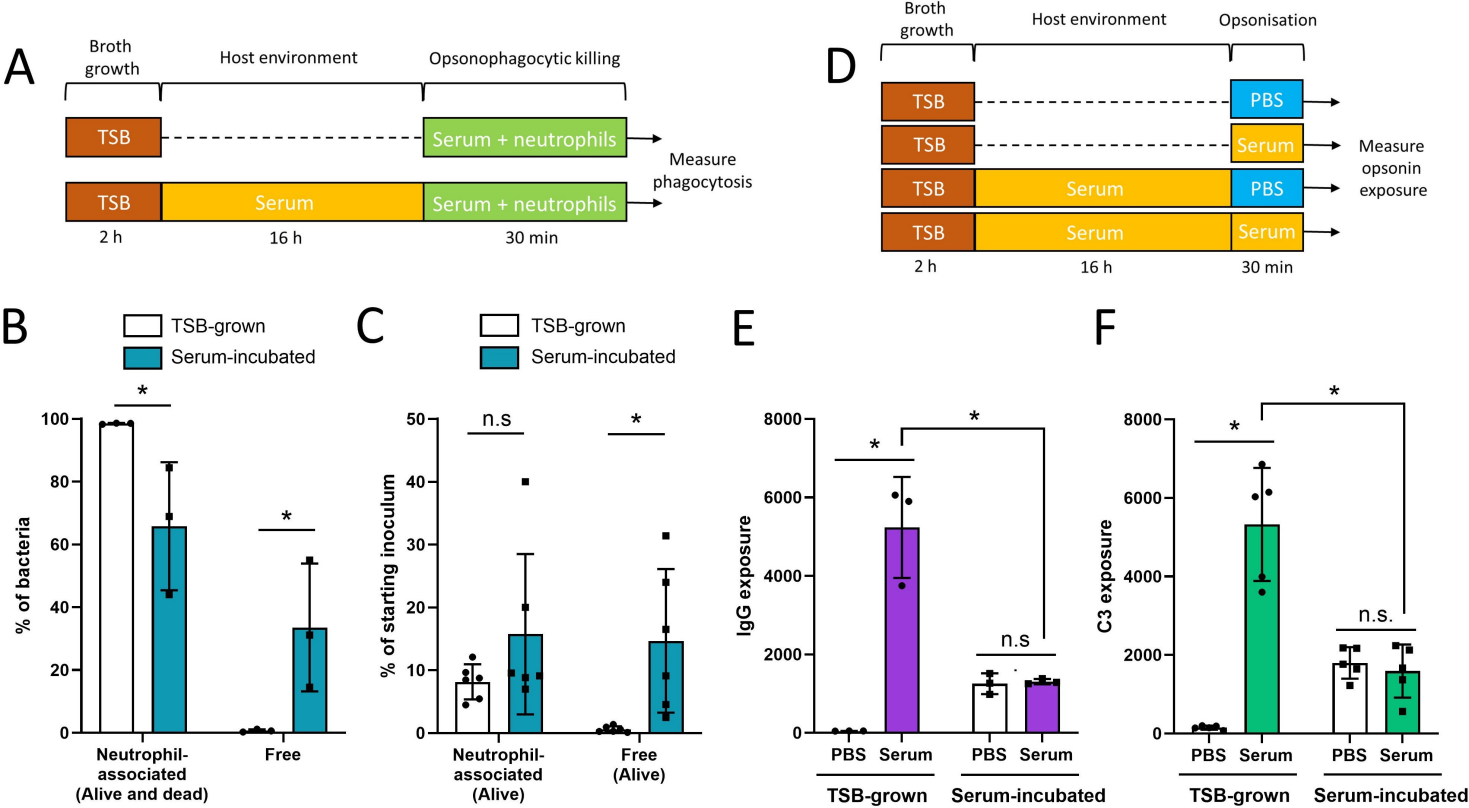
Incubation in human serum reduces opsonophagocytosis and surface-exposed IgG and complement. (**A**) *S. aureus* USA300 was grown in TSB or incubated in 100% serum, before incubation with purified human neutrophils for 30 min before the percentage of neutrophil-associated (phagocytosed) and non-neutrophil associated (free) bacteria were determined. To do this, two different approaches were used: flow cytometry (**B**) and CFU counts (**C**). (**D**) To assess opsonization, TSB-grown or serum-incubated *S. aureus* cells were incubated for 30 min with PBS only or PBS containing 10% serum before the levels of surface-exposed IgG (**E**) or C3 (**F**) was determined by flow cytometry. Data in panels B and C show values for each independent experiment, with bars representing the mean ± standard deviation. In E and F, each biological repeat is represented by the median fluorescence value of 10,000 bacterial cells. Data in panels B, C, E, and F were analyzed by two-way ANOVA with Sidak’s *post hoc* test (*P < 0.05; n.s, *P* ≥ 0.05 for the indicated comparisons).

We started by assessing whether the increase in survival of serum-incubated bacteria was due to impaired phagocytosis, using two distinct assays. Bacteria were grown in broth or incubated in serum, before being washed in PBS and then incubated with neutrophils in the presence of fresh serum to enable opsonization (Fig. 2A). The first phagocytosis assay was a flow cytometry-based approach that determined how many fluorescently labeled bacteria were associated (or not) with neutrophils (49) (Fig. S2). This revealed that the majority of both broth-grown and serum-incubated *S. aureus* were associated with neutrophils after 30-min incubation with the immune cells. However, while <3% of broth-grown bacteria remained unbound to neutrophils, >20% of serum-incubated bacteria were free (Fig. 2B). This finding was replicated in a second phagocytosis assay that measured the viability of free and neutrophil-associated bacteria (54), with >10% of serum-incubated *S. aureus* unphagocytosed compared with <1% of broth-grown *S. aureus* cells (Fig. 2C; Fig. S3) (55, 56). Using this second assay, we also found that serum-incubated stationary phase bacteria were phagocytosed less efficiently than TSB-grown stationary phase cells (Fig. S4).

Combined, these two assays demonstrated that serum-incubated *S. aureus* was significantly better at evading phagocytosis than broth-grown bacteria. An additional finding was that there were equal number of viable serum-incubated and TSB-grown *S. aureus* cells associated with neutrophils, regardless of growth phase (Fig. 2C; Fig. S4). This indicated that serum-incubated *S. aureus* cells were as susceptible to the microbicides produced by the neutrophils as broth-grown cells. Therefore, we concluded that the enhanced survival of serum-incubated bacteria compared with broth-grown bacteria (Fig. 1) was due to enhanced evasion of phagocytosis, rather than resistance to the antibacterial products of neutrophils.

To understand why more serum-incubated *S. aureus* cells were able to evade phagocytosis compared with TSB-grown cells, we first considered whether serum caused clumping of bacteria that precluded phagocytosis. However, using microscopy, we found that bacteria incubated in serum for 16 h did not form large clumps, relative to broth-grown *S. aureus*, which ruled out bacterial aggregation as an explanation for reduced phagocytosis (Fig. S5).

We then examined the degree of opsonization of bacteria by antibody and complement using western blotting. In keeping with previous work (57, 58), for this experiment, we used a mutant strain of USA300 lacking Spa and Sbi to avoid interference caused by these immunoglobulin-binding proteins (Fig. S6). TSB-grown or serum-incubated bacteria were washed in PBS and then incubated, or not, in fresh serum to enable opsonin binding as used in the opsonophagocytosis assays described above (Fig. 1A) before detection of bound antibody and complement component C3 (Fig. 2D).

Despite their reduced phagocytosis by neutrophils, there was more antibody and complement bound to serum-incubated cells than to TSB grown, suggesting that a lack of bound opsonins did not explain the immune evasion phenotype of serum-incubated bacteria (Fig. S7) (59).

To understand why serum-incubated cells had high levels of bound antibody and complement but low levels of phagocytosis, bacteria were prepared as described above for opsonophagocytosis assays and then the levels of surface-exposed antibody and the complement component C3 quantified using flow cytometry (Fig. 2D; Fig. S8). TSB-grown bacteria that had been exposed to PBS instead of serum acted as a negative control and confirmed that antibodies used in the assay did not bind non-specifically to *S. aureus* cells (Fig. 2E and F). We then showed that, as expected, TSB-grown bacteria that were incubated in human serum for 30 min were very strongly bound by both IgG and the complement component C3 (Fig. 2E and F).

Next, we examined serum-incubated bacteria and found that they had a significantly reduced level of exposed opsonins, compared with TSB-grown cells, regardless of whether they had been incubated in fresh serum for 30 min or not (Fig. 2E and F). Therefore, despite prolonged incubation in serum and high levels of bound antibody and complement (Fig. S7), serum-incubated cells had significantly reduced exposure of opsonins on their cell surface relative to TSB-grown bacteria that had been opsonized.

Taken together, these experiments revealed that serum-incubated bacteria are better able to survive exposure to neutrophils than broth-grown *S. aureus* because they are less likely to be phagocytosed, in keeping with the lower surface exposure of bound IgG and complement.

### Cell wall accumulation impairs opsonophagocytosis by concealing IgG bound to LTA and protein

**Fig 3:**
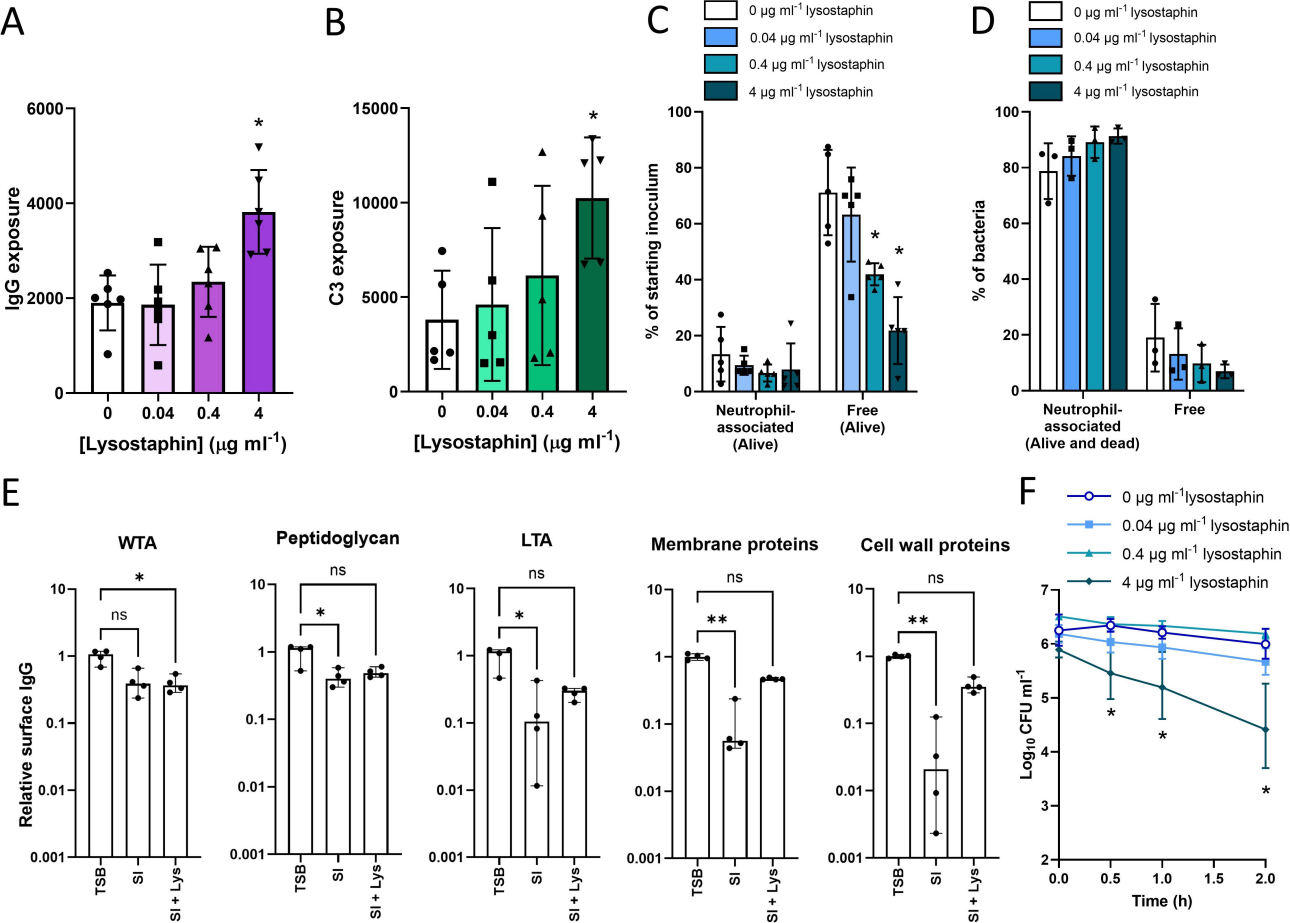
Cell wall accumulation impairs opsonophagocytosis by concealing bound opsonins. Cultures of *S. aureus* USA300 were incubated in 100% human serum for 16 h before treatment with indicated concentrations of lysostaphin for 20 min to partially digest the cell wall before levels of surface-exposed (**A**) IgG and (**B**) complement component C3 were determined by flow cytometry. In addition, these cells were incubated with purified human neutrophils for 30 min before the percentage of neutrophil-associated and non-neutrophil-associated (free) bacteria were determined by (**C**) CFU counts and (**D**) flow cytometry. (**E**) Bound IgG was eluted from the surface of *S. aureus* grown to exponential phase in broth and then incubated in serum for 30 min (TSB), or serum-incubated for 16 h (SI) or serum-incubated cells that were subsequently subjected to lysostaphin treatment (SI + Lys) as determined by ELISA. Eluted IgG was then assessed for binding to WTA, peptidoglycan, LTA, membrane-associated proteins, or cell wall-associated proteins. (**F**) Survival of serum-incubated lysostaphin-treated cells during a 2-h incubation with purified human neutrophils. Lysostaphin was washed away before incubation with neutrophils. Data in panels A–D represent the mean ± standard deviation of the indicated number of independent biological replicates. In panels A and B, each biological repeat is represented by the median fluorescence value of 10,000 bacterial events. Data in panels A and B were analyzed by one-way ANOVA with Dunnett’s *post hoc* test. Data in panels C, D, and F were analyzed by two-way ANOVA with Dunnett’s *post hoc* test (*P ≤ 0.05; lysostaphin-treated vs non-lysostaphin treated). Data in panel E represent the median ± 95% CI of four independent biological replicates and were analyzed by the Kruskal-Wallis test and Dunn’s *post hoc* test to establish statistically significant differences between groups (**P < 0.01; *P < 0.05; ns, *P* ≥ 0.05 for the indicated comparisons). Data in panel F represent the geometric mean ± geometric standard deviation of four independent experiments.

Since the cell envelope of *S. aureus* accumulates peptidoglycan and WTA during incubation in serum (37), we tested whether this concealed some of the bound antibody and complement. To do this, serum-incubated bacteria were subsequently incubated for 20 min with a range of sub-lethal concentrations of the enzyme lysostaphin, which cleaves peptidoglycan, to partially remove the cell wall. The lysostaphin was then removed by washing and bacterial viability was confirmed by CFU counts. This limited cell wall digestion resulted in a significant, dose-dependent increase in exposure of bound IgG and complement, demonstrating that some of the bound opsonins were concealed by the accumulation of cell wall polymers during incubation in serum (Fig. 3A and B).

We then tested whether the concealment of bound IgG by accumulated cell wall explained the reduced phagocytosis of serum-incubated bacteria relative to TSB-grown *S. aureus*. In keeping with increased IgG and complement exposure, limited lysostaphin treatment increased the phagocytosis of serum-incubated *S. aureus* by neutrophils (Fig. 3C and D).

Since human serum contains IgG that recognizes multiple *S. aureus* surface structures, we next sought to understand whether the reduced opsonization observed for serum-incubated bacteria was specific to a particular antibody target. Bacteria were grown in TSB and then incubated briefly in serum (30 min) or serum incubated (16 h). Surface-exposed IgG was then eluted from bacteria and assessed for its binding to each of the major surface structures by ELISA.

Serum-incubated bacteria had similar levels of anti-WTA IgG on their surface compared to exponential phase bacteria and only slightly lower levels of anti-peptidoglycan IgG (2.5-fold difference) (Fig. 3E). However, surface exposure of IgG targeting other surface structures was greatly reduced in serum-incubated compared to exponential phase cells, with anti-LTA IgG 9-fold lower, anti-membrane-associated proteins 18-fold lower, and anti-cell wall-associated proteins 48-fold lower (Fig. 3E). We did not examine anti-capsular polysaccharide antibodies in these assays as USA300 is deficient in this polymer (50). As such, the lower surface IgG exposure in serum-incubated cells compared to those in the exponential phase is primarily due to a loss of exposure of antibody bound to LTA and surface proteins.

Partial digestion of peptidoglycan using lysostaphin restored surface exposure of IgG bound to LTA and proteins to similar levels observed for TSB-grown bacteria (Fig. 3E). Therefore, accumulation of cell wall in serum-incubated bacteria preferentially conceals IgG bound to LTA and surface proteins, while anti-WTA and anti-peptidoglycan antibodies remain strongly exposed.

Finally, we showed that increasing opsonin exposure via partial lysostaphin digestion of the cell wall, with the enzyme washed away before incubation with immune cells, rendered serum-incubated staphylococci as susceptible to neutrophil-mediated killing as TSB-grown bacteria (Fig. 3F).

Taken together, the experiments described here demonstrate that serum-incubated *S. aureus* cells are bound by high levels of antibody and complement but the accumulation of cell wall conceals some of these bound opsonins, reducing phagocytosis and killing by neutrophils.

### Antibiotic-mediated inhibition of peptidoglycan accumulation maintains opsonin exposure and efficient opsonophagocytosis

To further test whether serum incubation reduced phagocytosis via cell wall-mediated concealment of bound opsonins, and to explore potential therapeutic approaches to enhance neutrophil-mediated killing, we first used the antibiotic fosfomycin to block the serum-induced accumulation of peptidoglycan, as we have done previously (37). This antibiotic targets MurA, which catalyzes the production of the peptidoglycan precursor UDP *N*-acetylmuramic acid in the cytoplasm (60). This inhibits peptidoglycan synthesis and prevents serum-induced cell wall thickening from occurring and has been used clinically in anti-staphylococcal combination therapies (37, 61).

**Fig 4:**
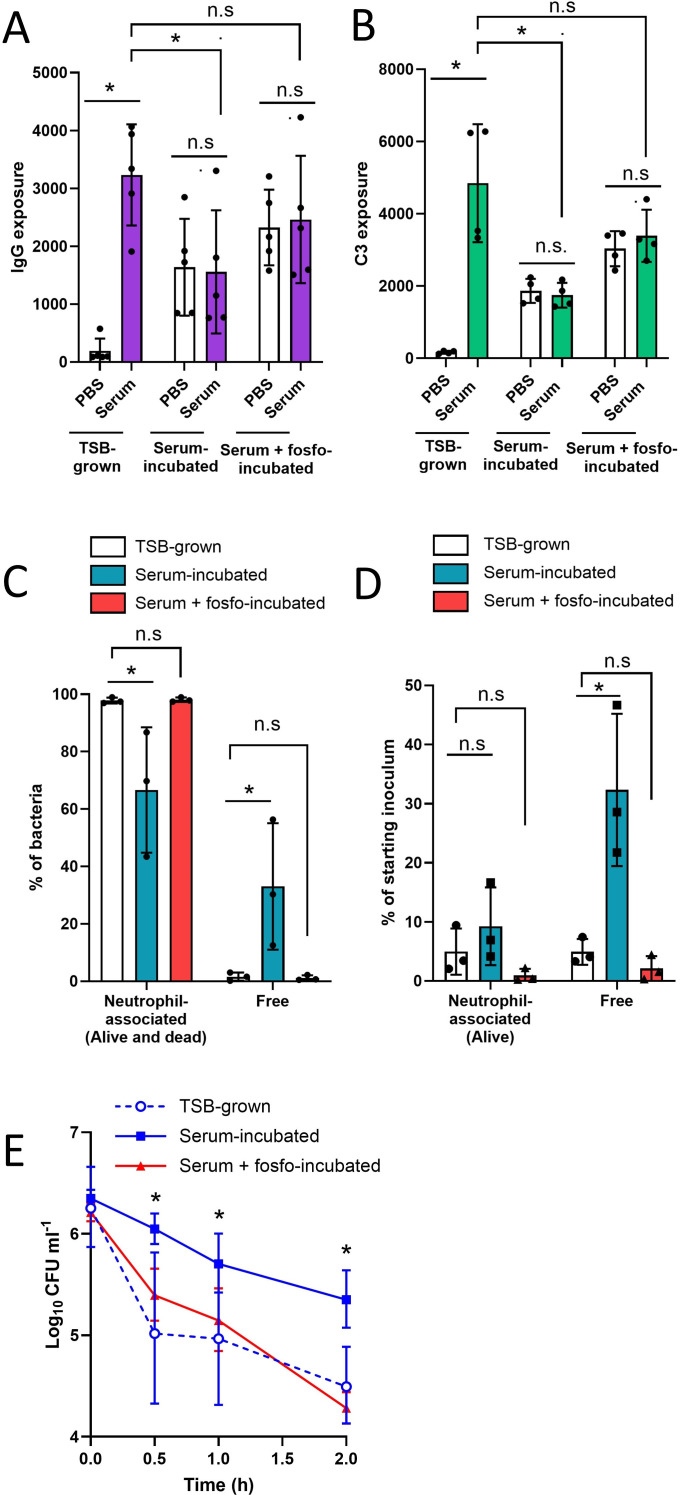
Antibiotic-mediated inhibition of cell wall accumulation maintains opsonin exposure and efficient opsonophagocytosis. *S. aureus* was TSB grown, incubated in 100% human serum (16 h) or incubated in 100% serum supplemented with fosfomycin (16 h) and subsequently incubated for 30 min with PBS or 10% serum before the levels of surface-exposed (**A**) IgG and (**B**) complement component C3 were determined by flow cytometry. In addition, these cells were incubated with purified human neutrophils for 30 min before the percentage of neutrophil-associated and non-neutrophil-associated (free) bacteria were determined by (**C**) flow cytometry and (**D**) CFU counts. (**E**) Survival of TSB-grown, serum-incubated, and serum + fosfomycin-incubated cultures during a 2-h incubation with purified human neutrophils. Data in panels A–D represent the geometric mean ± geometric standard deviation of the indicated number of independent biological replicates. Data in panel E are presented as the geometric mean ± geometric standard deviation of three independent experiments. In panels A and B, each biological repeat is represented by the median fluorescence value of 10,000 bacterial events. Data in panels A and B were analyzed by two-way ANOVA with Tukey’s *post hoc* test. Data in panels C–E were analyzed by two-way ANOVA with Dunnett’s *post hoc* test (*P < 0.05; n.s, *P* ≥ 0.05; comparisons are indicated in panels A–D and serum/serum +fosfomycin-incubated vs TSB grown at each time point in panel E).

As observed previously, serum-induced changes to *S. aureus* resulted in a significant reduction in opsonization, as determined by exposure of IgG and complement, relative to TSB-grown bacteria (Fig. 4A and B). However, the presence of fosfomycin in serum significantly reduced opsonin concealment, maintaining IgG and complement exposure at similar levels to that seen for TSB-grown bacteria (Fig. 4A and B).

Further analysis of IgG exposure confirmed that fosfomycin treatment preserved the exposure of IgG bound to all major surface structures relative to bacteria that had not been treated with the antibiotic (Fig. S9). Similar findings occurred with another inhibitor of peptidoglycan synthesis, oxacillin, which acts on penicillin-binding proteins (62), whereas antibiotics that targeted fatty acid biosynthesis (AFN-1252) or DNA gyrase (ciprofloxacin) did not increase IgG exposure relative to serum-incubated cells that had not been exposed to antibiotics (63, 64) (Fig. S9). Therefore, in support of our previous findings, we concluded that the accumulation of peptidoglycan during serum incubation significantly reduces the exposure of IgG bound to LTA and surface proteins.

The increased exposure of IgG and complement on the surface of bacteria incubated in serum containing fosfomycin, restored phagocytosis to levels seen with TSB-grown bacteria, as determined by both phagocytosis assays (Fig. 4C and D). Furthermore, bacteria that were incubated in serum with fosfomycin were killed by neutrophils as efficiently as TSB-grown bacteria, whereas serum-incubated bacteria not exposed to fosfomycin survived at significantly higher levels (Fig. 4E). In keeping with our analysis that AFN-1252 did not prevent IgG concealment during host adaptation, this antibiotic did not promote neutrophil-mediated killing of serum-incubated *S. aureus* (Fig. S10).

*S. aureus* anchors proteins to peptidoglycan via sortase enzymes (SrtA and SrtB)(65) and so we assessed whether the impact of fosfomycin on serum-incubated cells was due to interference with this process. However, serum-incubated mutants defective for SrtA or SrtB, which cannot anchor proteins to peptidoglycan, survived incubation with neutrophils as well as serum-incubated wild-type bacteria (Fig. S11).

Taken together, these findings provided additional evidence that serum-induced cell wall accumulation conceals opsonins bound to LTA and surface proteins, which, in turn, compromises phagocytosis and killing by neutrophils. They also indicate that the antibiotic fosfomycin, in addition to its antibacterial activity, may aid the clearance of infection by preventing the concealment of opsonins.

## DISCUSSION

The binding of antibodies and complement to the bacterial cell surface enables the detection and destruction of pathogens by phagocytic immune cells (10, 11, 13). The data presented here demonstrate that *S. aureus* can conceal a subset of bound opsonins via cell wall accumulation, significantly reducing opsonophagocytosis and killing by neutrophils, a previously unrecognized mechanism of immune evasion (Fig. S12).

Cell wall remodeling occurs in response to host stresses and protects against antibiotics and host defense peptides. It involves the accumulation of peptidoglycan and WTA (36, 37, 66), and it is therefore unsurprising that exposure to antibodies targeting these two polymers was least affected. By contrast, the exposure of antibodies bound to surface proteins and LTA was significantly reduced by serum-induced changes to the cell envelope, in keeping with their localization within the cell wall itself (67).

Previous work indicated that WTA can block antibodies from binding to antigens within the cell wall (68). Although WTA accumulates in the wall during serum incubation, it is currently unknown whether this contributes to the concealment of IgG bound to LTA or proteins. Unfortunately, since cell wall accumulation is dependent on D-alanine-labeled WTA (37), we could not use a WTA-deficient mutant to explore the role of this polymer in reducing opsonin exposure. However, our work did show that inhibition of peptidoglycan accumulation preserved antibody exposure and opsonophagocytic killing by neutrophils, demonstrating a key role in cell wall accumulation.

Host-induced peptidoglycan accumulation is due to a combination of peptidoglycan synthesis and inhibition of autolytic activity (37). Recent work has revealed that mutants lacking the Atl autolysin have defective surface exposure of staphylococcal surface proteins, inhibiting their recognition by reactive antibodies (69). Exposure of surface proteins was restored using enzymatic digestion of peptidoglycan, providing additional evidence that peptidoglycan accumulation can obscure surface antigens and prevent their detection by antibodies. However, the impact of concealment of surface proteins on opsonophagocytosis has not been investigated previously.

Several experimental vaccines have been developed based on surface proteins in an attempt to generate high serum titers of opsonizing antibodies. Unfortunately, despite very promising data from animal infection experiments, none of these vaccines have shown efficacy in humans (70, 71). Several plausible reasons for this discrepancy have been proposed, including the host specificity of staphylococcal immune evasion factors and previous staphylococcal infection directing the host toward non-protective immunity (14, 71–74).

Another difference between model infection of animals and natural infection in humans is the physiological state of the bacteria. For many animal infections, bacteria are grown in TSB immediately prior to administration into the animal and will therefore have high levels of multiple protein antigens exposed on their surface, which facilitates rapid opsonophagocytosis (47, 72, 75). By contrast, natural invasive infection typically begins with colonization of superficial sites such as an inserted IV catheter and so bacteria may be in a very different physiological state from those grown in laboratory media when they enter the bloodstream and are thus less well recognized by antibodies targeting surface proteins (73, 74, 76–78). As such, the addition of WTA as a vaccine antigen may provide a reasonable level of protection against bacteria that have accumulated cell walls and thus have reduced exposure to surface proteins.

Previous work has indicated that the thickened cell wall associated with vancomycin resistance reduces staphylococcal susceptibility to intracellular killing by neutrophils (79). However, our data did not show a difference in staphylococcal survival within neutrophils, with similar numbers of intracellular viable broth-grown and serum-incubated bacteria. Instead, the survival advantage of host adaptation appeared to be due to enhanced evasion of phagocytosis. It has also been reported that the staphylococcal cell envelope changes as bacteria enter the stationary phase, including increased cell wall thickness and reduced cell wall-associated protein content (36, 75). Therefore, we investigated whether the growth phase affected serum-induced changes to the propensity of *S. aureus* to evade phagocytosis. These experiments showed that serum incubation promoted evasion of phagocytosis of *S. aureus* grown to both exponential and stationary phases. As such, the growth phase at which bacteria encounter serum is irrelevant to subsequent cell wall remodeling and immune evasion.

We do not yet know whether these findings apply to other Gram-positive pathogens. However, since previous work has shown that serum triggers cell wall thickening in *Enterococcus faecalis* and viridans group streptococci, it is possible that our findings with *S. aureus* represent a broadly conserved mechanism of immune evasion (80).

Cell wall thickening in *S. aureus* is triggered by bacterial sensing of the host defense antimicrobial peptide LL-37 via the GraRS system (37). Since LL-37 is present in most tissues and among the earliest host responses to infection or trauma (81–83), we hypothesize that *S. aureus* has evolved to sense this AMP as an early indicator that it is subject to immune attack and provides an opportunity to employ defensive measures against the impending arrival of neutrophils. In support of this hypothesis, GraRS, the two-component system that detects LL-37, is activated in the early stages of staphylococcal skin colonization, while *S. aureus* mutants lacking GraRS are significantly less virulent than wild-type strains in invasive infection models (84–86).

In addition to protecting against opsonophagocytosis, LL-37 exposure triggers reduced susceptibility to the antibiotics daptomycin and vancomycin (37, 87), suggesting *S. aureus* employs strategies that are broadly protective against the twin threats of host immunity and antibiotic therapy. This is similar to our previous work showing that induction of the *S. aureus* general stress response regulated by the alternative sigma factor SigB can promote the survival of bacteria exposed to host defenses and various classes of antibiotics (88). Further support for the link between *S. aureus*-immune interactions and antibiotic tolerance comes from studies showing that oxidative stress conferred by phagocytic cells reduces staphylococcal susceptibility to antibiotics (89, 90).

While the immune response may compromise the efficacy of antibiotic therapy under certain circumstances, our study also highlights how antibiotics and the immune response can work synergistically by showing that fosfomycin blocked LL-37 induced cell wall thickening and thereby maintained exposure of bound opsonins, leading to efficient opsonophagocytic killing. In addition, previous work has suggested that fosfomycin also promotes the killing of *S. aureus* via enhanced production of the neutrophil oxidative burst (91). However, while we exposed *S. aureus* to fosfomycin in serum, this was removed by washing prior to incubation with neutrophils and thus does not explain the enhanced killing effect observed in our assays. This strongly suggests that there are at least two mechanisms by which fosfomycin and neutrophils synergize against *S. aureus* and a greater understanding of this may contribute to more effective therapeutic approaches that reduce the high incidence of relapsing or chronic staphylococcal infections (92).

In summary, we show that *S. aureus* cells are heavily opsonized upon initial exposure to serum. However, *S. aureus* responds to serum by accumulating peptidoglycan, which conceals bound opsonins, reducing phagocytosis and killing by neutrophils.

## MATERIALS AND METHODS

### Bacterial strains and growth conditions

Bacterial strains used in this study are shown in Table 1. Strains were grown at 37°C on tryptic soy agar (TSA) or in TSB with shaking (180 r.p.m.) supplemented with erythromycin (10 µg mL^−1^) or kanamycin (90 µg mL^−1^) when required.

**TABLE 1:** Strains used in this study

Strain	Relevant characteristics	Reference/source
USA300 JE2	LAC strain of the USA300 lineage of community-acquired MRSA cured of plasmids	50
JE2 *sbi*::Tn *spa*::*kan*	JE2 strain defective for immunoglobulin-binding proteins Spa and Sbi	This study
JE2 *srtA*::Tn	JE2 strain defective for sortase A, which anchors LPxTG motif-containing proteins to peptidoglycan	50
JE2 *srtB*::Tn	JE2 strain defective for sortase B, which anchors NPQTN motif-containing IsdC protein to peptidoglycan	50
SH1000	*rsbU*+ derivative of laboratory strain 8325-4	51
Col	Early hospital-associated MRSA isolate, commonly used in laboratory experiments	52
Newman	Clinical isolate, methicillin susceptible, used widely in animal and laboratory experiments	53

### Construction of strains

The JE2 *sbi*::Tn/*spa::kan* double mutant was constructed via transduction of the kanamycin resistance marker from Newman *spa::kan* (93) into the *sbi*::Tn mutant present in the NARSA transposon mutant library (50) using φ11.

### IgG Fc binding assay

The Fc portion of human Immunoglobulin G (1 mg, Abcam) was labeled with biotin (Thermo Scientific EZ-Link Sulfo-NHS-Biotin) before the removal of unbound biotin by dialysis. The labeled Fc portion was then incubated with PBS-washed bacterial cells for 30 min (10 µg protein and 10^9^ CFU *S*. *aureus* in 1 mL PBS). Unbound immunoglobulin fragment was removed by three rounds of washing with PBS before cells were incubated with streptavidin-alkaline phosphatase for 30 min. Cells were then washed with three rounds of PBS before incubation in 200 µL p-nitrophenol phosphate substrate solution for ELISA (Merck) for 10 min. Cells were then pelleted by centrifugation and the supernatant recovered and A_405_ determined.

### Generation of TSB-grown and serum-incubated bacterial cultures

To generate TSB-grown bacteria, cultures were grown for 16 h in TSB to stationary phase. These were then diluted to 10^7^ CFU mL^−1^ in fresh TSB and incubated for 2 h at 37°C until 10^8^ CFU mL^−1^ was reached. For some experiments, bacteria were used directly from stationary phase cultures.

To generate serum-incubated bacteria, broth-grown cultures were centrifuged (3,200 × *g* for 10 min), resuspended in an equal volume of human serum from human male AB plasma (Sigma), and incubated for 16 h at 37°C. As *S. aureus* is unable to replicate in human serum, these cultures were also at 10^8^ CFU mL^−1^ (37, 47, 48, 94). Where appropriate, serum was supplemented with a sub-lethal concentration of fosfomycin (64 µg mL^−1^), oxacillin (128 µg mL^−1^), ciprofloxacin (160 µg mL^−1^), or AFN-1252 (0.15 µg mL^−1^). These concentrations were chosen based on previous work that showed they were the maximum concentration that did not affect staphylococcal viability in serum (37).

Where appropriate, the cell walls of serum-incubated cultures were degraded by lysostaphin. To do this, 1 mL aliquots of serum-incubated bacteria were washed in PBS and resuspended in 1 mL PBS supplemented with indicated concentrations of lysostaphin (between 0.04 and 4 µg mL^−1^). Bacteria were incubated statically for 20 min at 37°C before being washed by 3 rounds of centrifugation in PBS.

### Purification of neutrophils

Neutrophils were extracted from 45 mL human blood and collected in heparin tubes to prevent coagulation. Blood (15 mL) was carefully layered over 30°C PolymorphPrep (20 mL) and centrifuged for 1 h at 500 × *g* to separate the different cell types. Neutrophils were collected, washed with Hanks balanced salt solution (HBSS), and adjusted to 5 × 10^6^ viable cells mL^−1^ in HBSS. Based on microscopy and trypan blue staining, we estimate purity at >95% and viability at >98%.

### Determination of bacterial killing by neutrophils and phagocytosis by CFU counts

Neutrophils were adjusted to 5 × 10^6^ cells mL^−1^ in HBSS supplemented with 10% human serum, 0.1 mM CaCl_2_, and 0.1 mM MgCl_2_. In the case of lysostaphin-treated bacteria, 10% serum was omitted from the HBSS.

TSB-grown/serum-incubated bacteria were generated as described above, washed three times in PBS, and then added to neutrophils at 5 × 10^6^ CFU mL^−1^. Tubes were incubated with end-over-end mixing at 37°C for 3 h and at each time point (0, 0.5, 1, and 2 h) aliquots were removed, serially diluted 10-fold in PBS with multiple rounds of pipetting to break up bacterial aggregates, and plated to enumerate CFU ml^−1^. Previous work has shown that this approach gives ~100% recovery of the inoculum when neutrophil-mediated killing is blocked, providing confidence that all viable bacteria are recovered, regardless of, for example, aggregate formation (49).

In addition, the number of phagocytosed/unphagocytosed bacteria was also enumerated at the 0.5 h time point. A 500 µL aliquot of the neutrophil/bacteria mixture was taken and centrifuged at 500 × *g* for 1 min to pellet the neutrophils, along with any neutrophil-associated bacteria. The supernatant (containing unphagocytosed bacteria) was serially diluted 10-fold in PBS and plated for CFU counts and the pellet was resuspended in 500 µL PBS, serially diluted 10-fold in PBS, and plated for CFU counts. The CFU mL^−1^ values of the pellet and the supernatant were divided by the CFU mL^−1^ of the starting inoculum to generate the percentage of CFU mL^−1^ neutrophil-associated and unphagocytosed, respectively.

To validate the experimental conditions used in this assay, two control experiments were run. First, bacteria that were prepared as described above were subjected to centrifugation and the CFU counts pre- and post-centrifugation were quantified to determine whether bacteria were pulled out of suspension under these conditions. Second, to understand whether bacteria associated with neutrophils were intracellular, bacteria were incubated with neutrophils as described above, before subsequent incubation with or without lysostaphin (40 µg mL^−1^) to kill extracellular bacteria. Neutrophils were then washed and CFU counts were determined before washing to remove the lytic enzyme. In a pilot experiment, neutrophils were lysed with Triton X-100 (0.1%) to determine whether this affected the recovery of CFU. However, this detergent was not used in other experiments.

### Measurement of phagocytosis by flow cytometry

To measure phagocytosis by flow cytometry neutrophils and bacteria were prepared as described above except that immediately before bacteria were added to the neutrophils, the bacteria were incubated with 10 µg mL^−1^ fluorescein isothiocyanate for 30 min at room temperature, and then washed three times in PBS.

As above, 5 × 10^6^ CFU mL^−1^ bacteria were added to 5 × 10^6^ cells mL^−1^ in HBSS supplemented with 10% human serum, 0.1 mM CaCl_2_ and 0.1 mM MgCl_2_. In the case of lysostaphin-treated bacteria, 10% serum was omitted from the HBSS. After a 30-min incubation at 37°C with end-over-end mixing in the dark, cultures were fixed by the addition of an equal volume of 4% paraformaldehyde (PFA). Samples were then analyzed by flow cytometry using an Amnis CellStream. Bacteria were detected using the 488 nm laser and at least 10,000 bacterial events were recorded. Events with FITC ≥2 × 10^3^ were counted as bacteria. Events with an FCS of ≥3,000 were counted as neutrophil-associated and <3000 were counted as free.

### Measurement of IgG and complement surface exposure by flow cytometry

TSB-grown and serum-incubated cultures were prepared as described above. A *spa/sbi* double mutant was used to prevent non-specific antibody binding. Aliquots (500 µL) were incubated for 30 min at room temperature in either PBS or 10% human serum. Samples were washed by three rounds of centrifugation in PBS (13,000 × *g* for 1 min) and blocked for 1 h in 4% BSA in PBS. Samples were washed once in PBS before IgG was detected with a 1:1,000 dilution of goat anti-human IgG antibody labeled with the BV421 fluorophore (Jackson ImmunoResearch) or C3 was detected with a 1:1,000 dilution of goat anti-human C3 F(ab′)_2_ labeled with FITC (Protos Immunoresearch). Antibody incubations were carried out statically for 1 h at room temperature in the dark. Samples were washed with PBS by three rounds of centrifugation (13,000 × *g* for 1 min) and fixed in 4% PFA. Samples were analyzed by flow cytometry using an Amnis CellStream. IgG was detected using the 405 nm laser and C3 using the 488 nm laser. At least 10,000 bacterial events were recorded and the median value was recorded.

### Measurement of IgG and complement by western blotting

Cultures of TSB-grown and serum-incubated bacteria (1 mL at 10^8^ CFU mL^−1^) were prepared as described above, washed by three rounds of centrifugation in PBS (13,000 × *g* for 1 min), and resuspended in 100 µL PBS. A *spa/sbi* double mutant was used to prevent non-specific antibody binding. Lysostaphin (10 µg mL^−1^) was added and bacteria were incubated statically for 1 h at 37°C. Sample buffer (187.5 mM Tris-HCl [pH 6.8], 6% SDS, 30% glycerol, 0.03% bromophenol blue, and 15% beta-mercaptoethanol; 50 µL) was added and samples were incubated at 95°C for 10 min before 15 µL was loaded onto 10% polyacrylamide gels. Gels were run in Tris-Glycine running buffer (25 mM Tris, 192 mM glycine, 0.1% SDS, pH 8.4) at 100 V for 10 min followed by 200 V for 50 min before being transferred onto PVDF membranes (10 V for 60 min). Membranes were blocked for 1 h at room temperature in 5% milk and 1% BSA in TBST. IgG was detected using 1:10,000 dilution of donkey anti-human IgG conjugated to HRP (Abcam) and C3 was detected by 1:5,000 dilution of rabbit anti-C3 (Abcam) followed by 1:10,000 dilution of goat anti-rabbit IgG conjugated to HRP (Abcam). Blots were developed using SuperSignal West Pico PLUS chemiluminescent substrate (Thermo Scientific) and imaged using the Bio-rad ChemiDoc MP imaging system.

### Characterization of IgG bound to cells

Bacteria were grown to exponential phase and incubated in serum for 30 min or 16 h, followed or not by partial cell wall digestion using lysostaphin as described above. Cells (10^8^) were washed three times in PBS before the bound antibody was eluted using 200 µL antibody elution buffer (Pierce) for 5 min. Cells were then removed by centrifugation and the eluted antibody solution was neutralized with 100 µL protein A binding buffer (Pierce).

To determine the binding ligands of bound antibodies, 10 µg purified cell surface components LTA (Sigma), WTA (37), peptidoglycan (37), membrane proteins (14), or cell wall proteins (14) were immobilized onto the wells of a Nunc Maxisorp ELISA plate by incubation at 4°C for 16 h. The remaining binding sites were blocked with PBS containing 3% bovine serum albumin before the addition of the eluted antibody samples (200 µL). Wells containing eluted antibodies were incubated at ambient temperature for 1 h, washed three times with PBS, and then 200 µL PBS containing anti-human antibodies conjugated to alkaline phosphatase (Abcam, 1:2,000 dilution) was added for 1 h. Wells were again washed three times with PBS and bound alkaline phosphatase quantified using a p-Nitrophenol phosphate substrate solution for ELISA (Merck) and A_405_ readings.

### Statistical analyses

CFU counts were log_10_ transformed and displayed as the geometric mean  ±  geometric standard deviations (95). Other data are displayed as the mean  ±  standard deviation or median ± 95% CI. For all experiments, three or more independent replicates were performed as indicated by individual data points. Data were analyzed by one-way ANOVA, two-way ANOVA, or Kruskal Wallis, with appropriate *post hoc* multiple comparison test as detailed in figure legends using GraphPad Prism (V8.0).

## Data Availability

All data supporting the findings of this study are available within the paper and its supplemental material.

## SUPPLEMENTAL MATERIAL

Supplemental material mbio.01643-24-s0001.docx Figures S1-S12.

ASM does not own the copyrights to Supplemental Material that may be linked to, or accessed through, an article. The authors have granted ASM a non-exclusive, world-wide license to publish the Supplemental Material files. Please contact the corresponding author directly for reuse.

## References

[1] Tong SYC, Davis JS, Eichenberger E, Holland TL, Fowler VG Jr. 2015. Staphylococcus aureus infections: epidemiology, pathophysiology, clinical manifestations, and management. Clin Microbiol Rev 28:603–661. doi:10.1128/CMR.00134-14 26016486 PMC4451395

[2] Yang Z, Wang J, Wang W, Zhang Y, Han L, Zhang Y, Nie X, Zhan S. 2015. Proportions of Staphylococcus aureus and methicillin-resistant Staphylococcus aureus in patients with surgical site infections in mainland China: a systematic review and meta-analysis. PLoS ONE 10:e0116079. doi:10.1371/journal.pone.0116079 25602284 PMC4300093

[3] Kuehl R, Morata L, Boeing C, Subirana I, Seifert H, Rieg S, Kern WV, Kim HB, Kim ES, Liao C-H, et al.. 2020. Defining persistent Staphylococcus aureus bacteraemia: secondary analysis of a prospective cohort study. Lancet Infect Dis 20:1409–1417. doi:10.1016/S1473-3099(20)30447-3 32763194

[4] Bergin SP, Holland TL, Fowler VG, Tong SYC. 2017. Sepsis, and infective endocarditis associated with Staphylococcus aureus. Curr Top Microbiol Immunol 409:263–296. doi:10.1007/82_2015_5001 26659121

[5] Fowler VG, Olsen MK, Corey GR, Woods CW, Cabell CH, Reller LB, Cheng AC, Dudley T, Oddone EZ. 2003. Clinical identifiers of complicated Staphylococcus aureus bacteremia. Arch Intern Med 163:2066–2072. doi:10.1001/archinte.163.17.2066 14504120

[6] Nguyen TH, Cheung GYC, Rigby KM, Kamenyeva O, Kabat J, Sturdevant DE, Villaruz AE, Liu R, Piewngam P, Porter AR, et al.. 2022. Rapid pathogen-specific recruitment of immune effector cells in the skin by secreted toxins. Nat Microbiol 7:62–72. doi:10.1038/s41564-021-01012-9 34873293 PMC8732318

[7] DeLeo FR, Diep BA, Otto M. 2009. Host defense and pathogenesis in Staphylococcus aureus infections. Infect Dis Clin North Am 23:17–34. doi:10.1016/j.idc.2008.10.003 19135914 PMC2748223

[8] Ellson CD, Davidson K, Ferguson GJ, O’Connor R, Stephens LR, Hawkins PT. 2006. Neutrophils from p40phox-/- mice exhibit severe defects in NADPH oxidase regulation and oxidant-dependent bacterial killing. J Exp Med 203:1927–1937. doi:10.1084/jem.20052069 16880254 PMC2118373

[9] Ha KP, Clarke RS, Kim GL, Brittan JL, Rowley JE, Mavridou DAI, Parker D, Clarke TB, Nobbs AH, Edwards AM. 2020. Staphylococcal DNA repair is required for infection. mBio 11:e02288-20. doi:10.1128/mBio.02288-20 33203752 PMC7683395

[10] Scribner DJ, Fahrney D. 1976. Neutrophil receptors for IgG and complement: their roles in the attachment and ingestion phases of phagocytosis. J Immunol 116:892–897. 1254970

[11] Wright AE, Douglas SR. 1904. An experimental investigation of the rôle of the blood fluids in connection with phagocytosis. Proc R Soc Lond:72357–72370. 10.1093/clinids/11.5.827 2682954

[12] Leuzzi R, Bodini M, Thomsen IP, Soldaini E, Bartolini E, Muzzi A, Clemente B, Galletti B, Manetti AGO, Giovani C, Censini S, Budroni S, Spensieri F, Borgogni E, Rossi Paccani S, Margarit I, Bagnoli F, Giudice GD, Creech CB. 2021. Dissecting the human response to Staphylococcus aureus systemic infections. Front Immunol 12:749432. doi:10.3389/fimmu.2021.749432 34819932 PMC8607524

[13] Guerra FE, Borgogna TR, Patel DM, Sward EW, Voyich JM. 2017. Epic immune battles of history: neutrophils vs Staphylococcus aureus. Front Cell Infect Microbiol 7:286. doi:10.3389/fcimb.2017.00286 28713774 PMC5491559

[14] Holtfreter S, Kolata J, Bröker BM. 2010. Towards the immune proteome of Staphylococcus aureus - The anti-S. aureus antibody response. Int J Med Microbiol 300:176–192. doi:10.1016/j.ijmm.2009.10.002 19889576

[15] Dryla A, Prustomersky S, Gelbmann D, Hanner M, Bettinger E, Kocsis B, Kustos T, Henics T, Meinke A, Nagy E. 2005. Comparison of antibody repertoires against Staphylococcus aureus in healthy individuals and in acutely infected patients. Clin Diagn Lab Immunol 12:387–398. doi:10.1128/CDLI.12.3.387-398.2005 15753252 PMC1065207

[16] Wergeland HI, Haaheim LR, Natås OB, Wesenberg F, Oeding P. 1989. Antibodies to staphylococcal peptidoglycan and its peptide epitopes, teichoic acid, and lipoteichoic acid in sera from blood donors and patients with staphylococcal infections. J Clin Microbiol 27:1286–1291. doi:10.1128/jcm.27.6.1286-1291.1989 2473994 PMC267543

[17] Kim MJ, Rah SY, An JH, Kurokawa K, Kim UH, Lee BL. 2015. Human anti-peptidoglycan-IgG-mediated opsonophagocytosis is controlled by calcium mobilization in phorbol myristate acetate-treated U937 cells. BMB Rep 48:36–41. doi:10.5483/bmbrep.2015.48.1.080 24856825 PMC4345640

[18] Jung DJ, An JH, Kurokawa K, Jung YC, Kim MJ, Aoyagi Y, Matsushita M, Takahashi S, Lee HS, Takahashi K, Lee BL. 2012. Specific serum Ig recognizing staphylococcal wall teichoic acid induces complement-mediated opsonophagocytosis against Staphylococcus aureus. J Immunol 189:4951–4959. doi:10.4049/jimmunol.1201294 23071283

[19] Lee JH, Kim NH, Winstel V, Kurokawa K, Larsen J, An JH, Khan A, Seong MY, Lee MJ, Andersen PS, Peschel A, Lee BL. 2015. Surface glycopolymers are crucial for in vitro anti-wall teichoic acid IgG-mediated complement activation and opsonophagocytosis of Staphylococcus aureus. Infect Immun 83:4247–4255. doi:10.1128/IAI.00767-15 26283333 PMC4598408

[20] Theilacker C, Kropec A, Hammer F, Sava I, Wobser D, Sakinc T, Codée JDC, Hogendorf WFJ, van der Marel GA, Huebner J. 2012. Protection against Staphylococcus aureus by antibody to the polyglycerolphosphate backbone of heterologous lipoteichoic acid. J Infect Dis 205:1076–1085. doi:10.1093/infdis/jis022 22362863

[21] de Jong NWM, vanKessel KPM, vanStrijp JAG. 2019. Immune evasion by Staphylococcus aureus. Microbiol Spectr 7. doi:10.1128/microbiolspec.GPP3-0061-2019 PMC11590434 30927347

[22] Thammavongsa V, Kim HK, Missiakas D, Schneewind O. 2015. Staphylococcal manipulation of host immune responses. Nat Rev Microbiol 13:529–543. doi:10.1038/nrmicro3521 26272408 PMC4625792

[23] Cruz AR, Boer M den, Strasser J, Zwarthoff SA, Beurskens FJ, de Haas CJC, Aerts PC, Wang G, de Jong RN, Bagnoli F, van Strijp JAG, van Kessel KPM, Schuurman J, Preiner J, Heck AJR, Rooijakkers SHM. 2021. Staphylococcal protein A inhibits complement activation by interfering with IgG hexamer formation. Proc Natl Acad Sci U S A 118:e2016772118. doi:10.1073/pnas.2016772118 33563762 PMC7896290

[24] Falugi F, Kim HK, Missiakas DM, Schneewind O. 2013. Role of protein A in the evasion of host adaptive immune responses by Staphylococcus aureus. mBio 4:e00575-13. doi:10.1128/mBio.00575-13 23982075 PMC3760252

[25] Zhang L, Jacobsson K, Vasi J, Lindberg M, Frykberg L. 1998. A second IgG-binding protein in Staphylococcus aureus. Microbiology (Reading) 144 ( Pt 4):985–991. doi:10.1099/00221287-144-4-985 9579072

[26] Smith EJ, Visai L, Kerrigan SW, Speziale P, Foster TJ. 2011. The Sbi protein is a multifunctional immune evasion factor of Staphylococcus aureus. Infect Immun 79:3801–3809. doi:10.1128/IAI.05075-11 21708997 PMC3165492

[27] de Haas CJC, Veldkamp KE, Peschel A, Weerkamp F, Van Wamel WJB, Heezius ECJM, Poppelier MJJG, Van Kessel KPM, van Strijp JAG. 2004. Chemotaxis inhibitory protein of Staphylococcus aureus, a bacterial antiinflammatory agent. J Exp Med 199:687–695. doi:10.1084/jem.20031636 14993252 PMC2213298

[28] Ko Y-P, Kuipers A, Freitag CM, Jongerius I, Medina E, van Rooijen WJ, Spaan AN, van Kessel KPM, Höök M, Rooijakkers SHM. 2013. Phagocytosis escape by a Staphylococcus aureus protein that connects complement and coagulation proteins at the bacterial surface. PLoS Pathog 9:e1003816. doi:10.1371/journal.ppat.1003816 24348255 PMC3861539

[29] Rooijakkers SHM, Ruyken M, Roos A, Daha MR, Presanis JS, Sim RB, van Wamel WJB, van Kessel KPM, van Strijp JAG. 2005. Immune evasion by a staphylococcal complement inhibitor that acts on C3 convertases. Nat Immunol 6:920–927. doi:10.1038/ni1235 16086019

[30] Rooijakkers SHM, Ruyken M, van Roon J, van Kessel KPM, van Strijp JAG, van Wamel WJB. 2006. Early expression of SCIN and CHIPS drives instant immune evasion by Staphylococcus aureus. Cell Microbiol 8:1282–1293. doi:10.1111/j.1462-5822.2006.00709.x 16882032

[31] Howden BP, Giulieri SG, Wong Fok Lung T, Baines SL, Sharkey LK, Lee JYH, Hachani A, Monk IR, Stinear TP. 2023. Staphylococcus aureus host interactions and adaptation. Nat Rev Microbiol 21:380–395. doi:10.1038/s41579-023-00852-y 36707725 PMC9882747

[32] Pidgeon SE, Pires MM. 2017. Cell wall remodeling of Staphylococcus aureus in live Caenorhabditis elegans. Bioconjug Chem 28:2310–2315. doi:10.1021/acs.bioconjchem.7b00363 28737895

[33] Sobral R, Tomasz A. 2019. The staphylococcal cell wall. Microbiol Spectr 7. doi:10.1128/microbiolspec.gpp3-0068-2019 PMC10957225 31322105

[34] Salamaga B, Kong L, Pasquina-Lemonche L, Lafage L, von Und Zur Muhlen M, Gibson JF, Grybchuk D, Tooke AK, Panchal V, Culp EJ, Tatham E, O’Kane ME, Catley TE, Renshaw SA, Wright GD, Plevka P, Bullough PA, Han A, Hobbs JK, Foster SJ. 2021. Demonstration of the role of cell wall homeostasis in Staphylococcus aureus growth and the action of bactericidal antibiotics. Proc Natl Acad Sci U S A 118:e2106022118. doi:10.1073/pnas.2106022118 34716264 PMC8612353

[35] Monteiro JM, Fernandes PB, Vaz F, Pereira AR, Tavares AC, Ferreira MT, Pereira PM, Veiga H, Kuru E, VanNieuwenhze MS, Brun YV, Filipe SR, Pinho MG. 2015. Cell shape dynamics during the staphylococcal cell cycle. Nat Commun 6:8055. doi:10.1038/ncomms9055 26278781 PMC4557339

[36] Sutton JAF, Carnell OT, Lafage L, Gray J, Biboy J, Gibson JF, Pollitt EJG, Tazoll SC, Turnbull W, Hajdamowicz NH, Salamaga B, Pidwill GR, Condliffe AM, Renshaw SA, Vollmer W, Foster SJ. 2021. Staphylococcus aureus cell wall structure and dynamics during host-pathogen interaction. PLoS Pathog 17:e1009468. doi:10.1371/journal.ppat.1009468 33788901 PMC8041196

[37] Ledger EVK, Mesnage S, Edwards AM. 2022. Human serum triggers antibiotic tolerance in Staphylococcus aureus. Nat Commun 13:2041. doi:10.1038/s41467-022-29717-3 35440121 PMC9018823

[38] Ellington JK, Harris M, Hudson MC, Vishin S, Webb LX, Sherertz R. 2006. Intracellular Staphylococcus aureus and antibiotic resistance: implications for treatment of staphylococcal osteomyelitis. J Orthop Res 24:87–93. doi:10.1002/jor.20003 16419973

[39] Raineri EJM, Yedavally H, Salvati A, van Dijl JM. 2020. Time-resolved analysis of Staphylococcus aureus invading the endothelial barrier. Virulence 11:1623–1639. doi:10.1080/21505594.2020.1844418 33222653 PMC7714425

[40] Reed P, Atilano ML, Alves R, Hoiczyk E, Sher X, Reichmann NT, Pereira PM, Roemer T, Filipe SR, Pereira-Leal JB, Ligoxygakis P, Pinho MG. 2015. Staphylococcus aureus survives with a minimal peptidoglycan synthesis machine but sacrifices virulence and antibiotic resistance. PLoS Pathog 11:e1004891. doi:10.1371/journal.ppat.1004891 25951442 PMC4423922

[41] Hines KM, Alvarado G, Chen X, Gatto C, Pokorny A, Alonzo F III, Wilkinson BJ, Xu L. 2020. Lipidomic and ultrastructural characterization of the cell envelope of Staphylococcus aureus grown in the presence of human serum. mSphere 5:e00339–20. doi:10.1128/mSphere.00339-20 32554713 PMC7300354

[42] Yarwood JM, McCormick JK, Paustian ML, Kapur V, Schlievert PM. 2002. Repression of the Staphylococcus aureus accessory gene regulator in serum and in vivo. J Bacteriol 184:1095–1101. doi:10.1128/jb.184.4.1095-1101.2002 11807070 PMC134826

[43] James EH, Edwards AM, Wigneshweraraj S. 2013. Transcriptional downregulation of agr expression in Staphylococcus aureus during growth in human serum can be overcome by constitutively active mutant forms of the sensor kinase AgrC. FEMS Microbiol Lett 349:153–162. doi:10.1111/1574-6968.12309 24164684 PMC4274972

[44] Manifold-Wheeler BC, Elmore BO, Triplett KD, Castleman MJ, Otto M, Hall PR. 2016. Serum lipoproteins are critical for pulmonary innate defense against Staphylococcus aureus quorum sensing. J Immunol 196:328–335. doi:10.4049/jimmunol.1501835 26608923 PMC4685034

[45] Peterson MM, Mack JL, Hall PR, Alsup AA, Alexander SM, Sully EK, Sawires YS, Cheung AL, Otto M, Gresham HD. 2008. Apolipoprotein B Is an innate barrier against invasive Staphylococcus aureus infection. Cell Host Microbe 4:555–566. doi:10.1016/j.chom.2008.10.001 19064256 PMC2639768

[46] Hall PR, Elmore BO, Spang CH, Alexander SM, Manifold-Wheeler BC, Castleman MJ, Daly SM, Peterson MM, Sully EK, Femling JK, Otto M, Horswill AR, Timmins GS, Gresham HD. 2013. Nox2 modification of LDL is essential for optimal apolipoprotein B-mediated control of agr type III Staphylococcus aureus quorum-sensing. PLoS Pathog 9:e1003166. doi:10.1371/journal.ppat.1003166 23459693 PMC3573103

[47] Malachowa N, Whitney AR, Kobayashi SD, Sturdevant DE, Kennedy AD, Braughton KR, Shabb DW, Diep BA, Chambers HF, Otto M, DeLeo FR. 2011. Global changes in Staphylococcus aureus gene expression in human blood. PLoS One 6:e18617. doi:10.1371/journal.pone.0018617 21525981 PMC3078114

[48] Cybulska J, Jeljaszewicz J. 1966. Bacteriostatic activity of serum against staphylococci. J Bacteriol 91:953–962. doi:10.1128/jb.91.3.953-962.1966 5929770 PMC315984

[49] Painter KL, Hall A, Ha KP, Edwards AM. 2017. The electron transport chain sensitizes Staphylococcus aureus and Enterococcus faecalis to the oxidative burst. Infect Immun 85:e00659-17. doi:10.1128/IAI.00659-17 28993457 PMC5695107

[50] Fey PD, Endres JL, Yajjala VK, Widhelm TJ, Boissy RJ, Bose JL, Bayles KW. 2013. A genetic resource for rapid and comprehensive phenotype screening of nonessential Staphylococcus aureus genes. mBio 4:e00537-12. doi:10.1128/mBio.00537-12 23404398 PMC3573662

[51] Horsburgh MJ, Aish JL, White IJ, Shaw L, Lithgow JK, Foster SJ. 2002. sigmaB modulates virulence determinant expression and stress resistance: characterization of a functional rsbU strain derived from Staphylococcus aureus 8325-4. J Bacteriol 184:5457–5467. doi:10.1128/JB.184.19.5457-5467.2002 12218034 PMC135357

[52] Gill SR, Fouts DE, Archer GL, Mongodin EF, Deboy RT, Ravel J, Paulsen IT, Kolonay JF, Brinkac L, Beanan M, et al.. 2005. Insights on evolution of virulence and resistance from the complete genome analysis of an early methicillin-resistant Staphylococcus aureus strain and a biofilm-producing methicillin-resistant Staphylococcus epidermidis strain. J Bacteriol 187:2426–2438. doi:10.1128/JB.187.7.2426-2438.2005 15774886 PMC1065214

[53] Baba T, Bae T, Schneewind O, Takeuchi F, Hiramatsu K. 2008. Genome sequence of Staphylococcus aureus strain Newman and comparative analysis of staphylococcal genomes: polymorphism and evolution of two major pathogenicity islands. J Bacteriol 190:300–310. doi:10.1128/JB.01000-07 17951380 PMC2223734

[54] Nagl M, Kacani L, Müllauer B, Lemberger EM, Stoiber H, Sprinzl GM, Schennach H, Dierich MP. 2002. Phagocytosis and killing of bacteria by professional phagocytes and dendritic cells. Clin Diagn Lab Immunol 9:1165–1168. doi:10.1128/cdli.9.6.1165-1168.2002 12414745 PMC130096

[55] Easmon CS, Lanyon H, Cole PJ. 1978. Use of lysostaphin to remove cell-adherent staphylococci during in vitro assays of phagocyte function. Br J Exp Pathol 59:381–385. 708586 PMC2041367

[56] Simon GL, Miller HG, Borenstein DG. 1983. Synovial fluid inhibits killing of Staphylococcus aureus by neutrophils. Infect Immun 40:1004–1010. doi:10.1128/iai.40.3.1004-1010.1983 6303954 PMC348150

[57] Wonfor T, Li S, Dunphy RW, Macpherson A, van den Elsen J, Laabei M. 2022. Novel method for detecting complement C3 deposition on Staphylococcus aureus. Sci Rep 12:15766. doi:10.1038/s41598-022-20098-7 36130996 PMC9492775

[58] Boero E, Brinkman I, Juliet T, van Yperen E, van Strijp JAG, Rooijakkers SHM, van Kessel KPM. 2021. Use of flow cytometry to evaluate phagocytosis of Staphylococcus aureus by human neutrophils. Front Immunol 12:635825. doi:10.3389/fimmu.2021.635825 33679791 PMC7934835

[59] Cunnion KM, Hair PS, Buescher ES. 2004. Cleavage of complement C3b to iC3b on the surface of Staphylococcus aureus is mediated by serum complement factor I. Infect Immun 72:2858–2863. doi:10.1128/IAI.72.5.2858-2863.2004 15102797 PMC387901

[60] Silver LL. 2017. Fosfomycin: mechanism and resistance. Cold Spring Harb Perspect Med 7:a025262. doi:10.1101/cshperspect.a025262 28062557 PMC5287057

[61] Pujol M, Miró J-M, Shaw E, Aguado J-M, San-Juan R, Puig-Asensio M, Pigrau C, Calbo E, Montejo M, Rodriguez-Álvarez R, et al.. 2021. Daptomycin plus fosfomycin versus daptomycin alone for methicillin-resistant Staphylococcus aureus bacteremia and endocarditis: a randomized clinical trial. Clin Infect Dis 72:1517–1525. doi:10.1093/cid/ciaa1081 32725216 PMC8096235

[62] Wright AJ, Wilkowske CJ. 1991. The penicillins. Mayo Clin Proc 66:1047–1063. doi:10.1016/s0025-6196(12)61730-3 1921489

[63] Sanders CC. 1988. Ciprofloxacin: in vitro activity, mechanism of action, and resistance. Rev Infect Dis 10:516–527. doi:10.1093/clinids/10.3.516 3293157

[64] Karlowsky JA, Kaplan N, Hafkin B, Hoban DJ, Zhanel GG. 2009. AFN-1252, a FabI inhibitor, demonstrates a Staphylococcus-specific spectrum of activity. Antimicrob Agents Chemother 53:3544–3548. doi:10.1128/AAC.00400-09 19487444 PMC2715641

[65] Schneewind O, Mihaylova-Petkov D, Model P. 1993. Cell wall sorting signals in surface proteins of gram-positive bacteria. EMBO J 12:4803–4811. doi:10.1002/j.1460-2075.1993.tb06169.x 8223489 PMC413927

[66] Douglas EJA, Palk N, Brignoli T, Altwiley D, Boura M, Laabei M, Recker M, Cheung GYC, Liu R, Hsieh RC, Otto M, O’Brien E, McLoughlin RM, Massey RC. 2023. Extensive remodelling of the cell wall during the development of Staphylococcus aureus bacteraemia. Elife 12:RP87026. doi:10.7554/eLife.87026 37401629 PMC10328498

[67] Rajagopal M, Walker S. 2017. Envelope structures of gram-positive bacteria. Curr Top Microbiol Immunol 404:1–44. doi:10.1007/82_2015_5021 26919863 PMC5002265

[68] Gautam S, Kim T, Lester E, Deep D, Spiegel DA. 2016. Wall teichoic acids prevent antibody binding to epitopes within the cell wall of Staphylococcus aureus. ACS Chem Biol 11:25–30. doi:10.1021/acschembio.5b00439 26502318 PMC5592732

[69] Leonard AC, Goncheva MI, Gilbert SE, Shareefdeen H, Petrie LE, Thompson LK, Khursigara CM, Heinrichs DE, Cox G. 2023. Autolysin-mediated peptidoglycan hydrolysis is required for the surface display of Staphylococcus aureus cell wall-anchored proteins. Proc Natl Acad Sci U S A 120:e2301414120. doi:10.1073/pnas.2301414120 36920922 PMC10041135

[70] Miller LS, Fowler VG, Shukla SK, Rose WE, Proctor RA. 2020. Development of a vaccine against Staphylococcus aureus invasive infections: evidence based on human immunity, genetics and bacterial evasion mechanisms. FEMS Microbiol Rev 44:123–153. doi:10.1093/femsre/fuz030 31841134 PMC7053580

[71] Tsai C-M, Caldera JR, Hajam IA, Chiang AWT, Tsai C-H, Li H, Díez ML, Gonzalez C, Trieu D, Martins GA, Underhill DM, Arditi M, Lewis NE, Liu GY. 2022. Non-protective immune imprint underlies failure of Staphylococcus aureus IsdB vaccine. Cell Host & Microbe 30:1163–1172. doi:10.1016/j.chom.2022.06.006 35803276 PMC9378590

[72] Kim HK, Missiakas D, Schneewind O. 2014. Mouse models for infectious diseases caused by Staphylococcus aureus. J Immunol Methods 410:88–99. doi:10.1016/j.jim.2014.04.007 24769066 PMC6211302

[73] Luna BM, Nielsen TB, Cheng B, Pantapalangkoor P, Yan J, Boyle-Vavra S, Bruhn KW, Montgomery C, Spellberg B, Daum R. 2019. Vaccines targeting Staphylococcus aureus skin and bloodstream infections require different composition. PLoS ONE 14:e0217439. doi:10.1371/journal.pone.0217439 31181086 PMC6557488

[74] Clegg J, Soldaini E, McLoughlin RM, Rittenhouse S, Bagnoli F, Phogat S. 2021. Staphylococcus aureus vaccine research and development: the past, present and future, including novel therapeutic strategies. Front Immunol 12:705360. doi:10.3389/fimmu.2021.705360 34305945 PMC8294057

[75] Foster TJ. 2019. Surface proteins of Staphylococcus aureus. Microbiol Spectr 7. doi:10.1128/microbiolspec.GPP3-0046-2018 PMC10957221 31267926

[76] Marrie TJ, Costerton JW. 1984. Scanning and transmission electron microscopy of in situ bacterial colonization of intravenous and intraarterial catheters. J Clin Microbiol 19:687–693. doi:10.1128/jcm.19.5.687-693.1984 6429190 PMC271156

[77] Hetem DJ, de Ruiter SC, Buiting AGM, Kluytmans JAJW, Thijsen SF, Vlaminckx BJM, Wintermans RGF, Bonten MJM, Ekkelenkamp MB. 2011. Preventing Staphylococcus aureus bacteremia and sepsis in patients with Staphylococcus aureus colonization of intravascular catheters: a retrospective multicenter study and meta-analysis. Medicine (Baltimore) 90:284–288. doi:10.1097/MD.0b013e31822403e9 21694650

[78] Minejima E, Mai N, Bui N, Mert M, Mack WJ, She RC, Nieberg P, Spellberg B, Wong-Beringer A. 2020. Defining the breakpoint duration of Staphylococcus aureus bacteremia predictive of poor outcomes. Clin Infect Dis 70:566–573. doi:10.1093/cid/ciz257 30949675 PMC7768749

[79] Falcón R, Martínez A, Albert E, Madrid S, Oltra R, Giménez E, Soriano M, Vinuesa V, Gozalbo D, Gil ML, Navarro D. 2016. High vancomycin MICs within the susceptible range in Staphylococcus aureus bacteraemia isolates are associated with increased cell wall thickness and reduced intracellular killing by human phagocytes. Int J Antimicrob Agents 47:343–350. doi:10.1016/j.ijantimicag.2016.01.014 27056298

[80] Tickle ARH, Ledger EVK, Edwards AM. 2022. Human serum-induces daptomycin tolerance in Enterococcus faecalis and viridans group streptococci. Microbiology (Reading) 168:12. doi:10.1099/mic.0.001282 36748501

[81] Ong PY, Ohtake T, Brandt C, Strickland I, Boguniewicz M, Ganz T, Gallo RL, Leung DYM. 2002. Endogenous antimicrobial peptides and skin infections in atopic dermatitis. N Engl J Med 347:1151–1160. doi:10.1056/NEJMoa021481 12374875

[82] Nizet V, Ohtake T, Lauth X, Trowbridge J, Rudisill J, Dorschner RA, Pestonjamasp V, Piraino J, Huttner K, Gallo RL. 2001. Innate antimicrobial peptide protects the skin from invasive bacterial infection. Nature 414:454–457. doi:10.1038/35106587 11719807

[83] Dorschner RA, Pestonjamasp VK, Tamakuwala S, Ohtake T, Rudisill J, Nizet V, Agerberth B, Gudmundsson GH, Gallo RL. 2001. Cutaneous injury induces the release of cathelicidin antimicrobial peptides active against group A Streptococcus. J Invest Dermatol 117:91–97. doi:10.1046/j.1523-1747.2001.01340.x 11442754

[84] Burian M, Plange J, Schmitt L, Kaschke A, Marquardt Y, Huth L, Baron JM, Hornef MW, Wolz C, Yazdi AS. 2021. Adaptation of Staphylococcus aureus to the human skin environment identified using an ex vivo tissue model. Front. Microbiol 12:728989. doi:10.3389/fmicb.2021.728989 34621255 PMC8490888

[85] Kraus D, Herbert S, Kristian SA, Khosravi A, Nizet V, Götz F, Peschel A. 2008. The GraRS regulatory system controls Staphylococcus aureus susceptibility to antimicrobial host defenses. BMC Microbiol 8:85. doi:10.1186/1471-2180-8-85 18518949 PMC2430206

[86] Cheung AL, Cho J, Bayer AS, Yeaman MR, Xiong YQ, Donegan NP, Mikheyeva IV, Lee GY, Yang S-J. 2021. Role of the Staphylococcus aureus extracellular loop of GraS in resistance to distinct human defense peptides in PMN and invasive cardiovascular infections. Infect Immun 89:e0034721. doi:10.1128/IAI.00347-21 34227840 PMC8445198

[87] Friberg C, Haaber JK, Vestergaard M, Fait A, Perrot V, Levin BR, Ingmer H. 2020. Human antimicrobial peptide, LL-37, induces non-inheritable reduced susceptibility to vancomycin in Staphylococcus aureus. Sci Rep 10:13121. doi:10.1038/s41598-020-69962-4 32753585 PMC7403302

[88] Ranganathan N, Johnson R, Edwards AM. 2020. The general stress response of Staphylococcus aureus promotes tolerance of antibiotics and survival in whole human blood. Microbiology (Reading) 166:1088–1094. doi:10.1099/mic.0.000983 33095698 PMC7723259

[89] Rowe SE, Wagner NJ, Li L, Beam JE, Wilkinson AD, Radlinski LC, Zhang Q, Miao EA, Conlon BP. 2020. Reactive oxygen species induce antibiotic tolerance during systemic Staphylococcus aureus infection. Nat Microbiol 5:282–290. doi:10.1038/s41564-019-0627-y 31819212 PMC6992501

[90] Beam JE, Rowe SE, Conlon BP. 2021. Shooting yourself in the foot: how immune cells induce antibiotic tolerance in microbial pathogens. PLoS Pathog 17:e1009660. doi:10.1371/journal.ppat.1009660 34293056 PMC8297873

[91] Shen F, Tang X, Cheng W, Wang Y, Wang C, Shi X, An Y, Zhang Q, Liu M, Liu B, Yu L. 2016. Fosfomycin enhances phagocyte-mediated killing of Staphylococcus aureus by extracellular traps and reactive oxygen species. Sci Rep 6:19262. doi:10.1038/srep19262 26778774 PMC4726045

[92] Berti A, Rose W, Nizet V, Sakoulas G. 2020. Antibiotics and innate immunity: a cooperative effort toward the successful treatment of infections. Open Forum Infect Dis 7:ofaa302. doi:10.1093/ofid/ofaa302 32818143 PMC7423293

[93] Higgins J, Loughman A, van Kessel KPM, van Strijp JAG, Foster TJ. 2006. Clumping factor A of Staphylococcus aureus inhibits phagocytosis by human polymorphonuclear leucocytes. FEMS Microbiol Lett 258:290–296. doi:10.1111/j.1574-6968.2006.00229.x 16640587

[94] Ehrenkranz NJ, Elliott DF, Zarco R. 1971. Serum bacteriostasis of Staphylococcus aureus. Infect Immun 3:664–670. doi:10.1128/iai.3.5.664-670.1971 16558034 PMC416214

[95] Olsen CH. 2003. Review of the use of statistics in infection and immunity. Infect Immun 71:6689–6692. doi:10.1128/IAI.71.12.6689-6692.2003 14638751 PMC308915

