# Supplementary material for: Host-induced cell wall remodeling impairs opsonophagocytosis of Staphylococcus aureus by neutrophils
Source: mBio. 2024 Jul 23;15(8):e01643-24. doi: 10.1128/mbio.01643-24 (PMC11323798; doi:10.1128/mbio.01643-24)
Supplement: Supplemental material — Figures S1-S12. [file mbio.01643-24-s0001.docx]

**Host-induced cell wall remodelling impairs opsonophagocytosis of *Staphylococcus aureus* by neutrophils**

Elizabeth V. K. Ledger^1^ and Andrew M. Edwards^1*^

^1^ Centre for Bacterial Resistance Biology, Imperial College London, Armstrong Rd, London, SW7 2AZ, UK.

**Supplementary data file**

Supplementary Figures S1-S12.

**Supplementary figure S1. Reduced susceptibility of serum-incubated *S. aureus* to killing by neutrophils is not dependent on growth phase.** Stationary phase bacteria were incubated in 100% human serum for 16 h, or not, before incubation with neutrophils and survival measured after 2 h. Similar to exponential phase bacteria, serum-incubated *S. aureus* survived at significantly higher levels than bacteria that had not been incubated in serum. Bars show the geometric mean of 4 independent experiments, with each data point indicated with a circle. Error bars indicate the geometric standard deviation. Data were analysed by paired two-tailed student’s t-test. ** p = 0.0023.


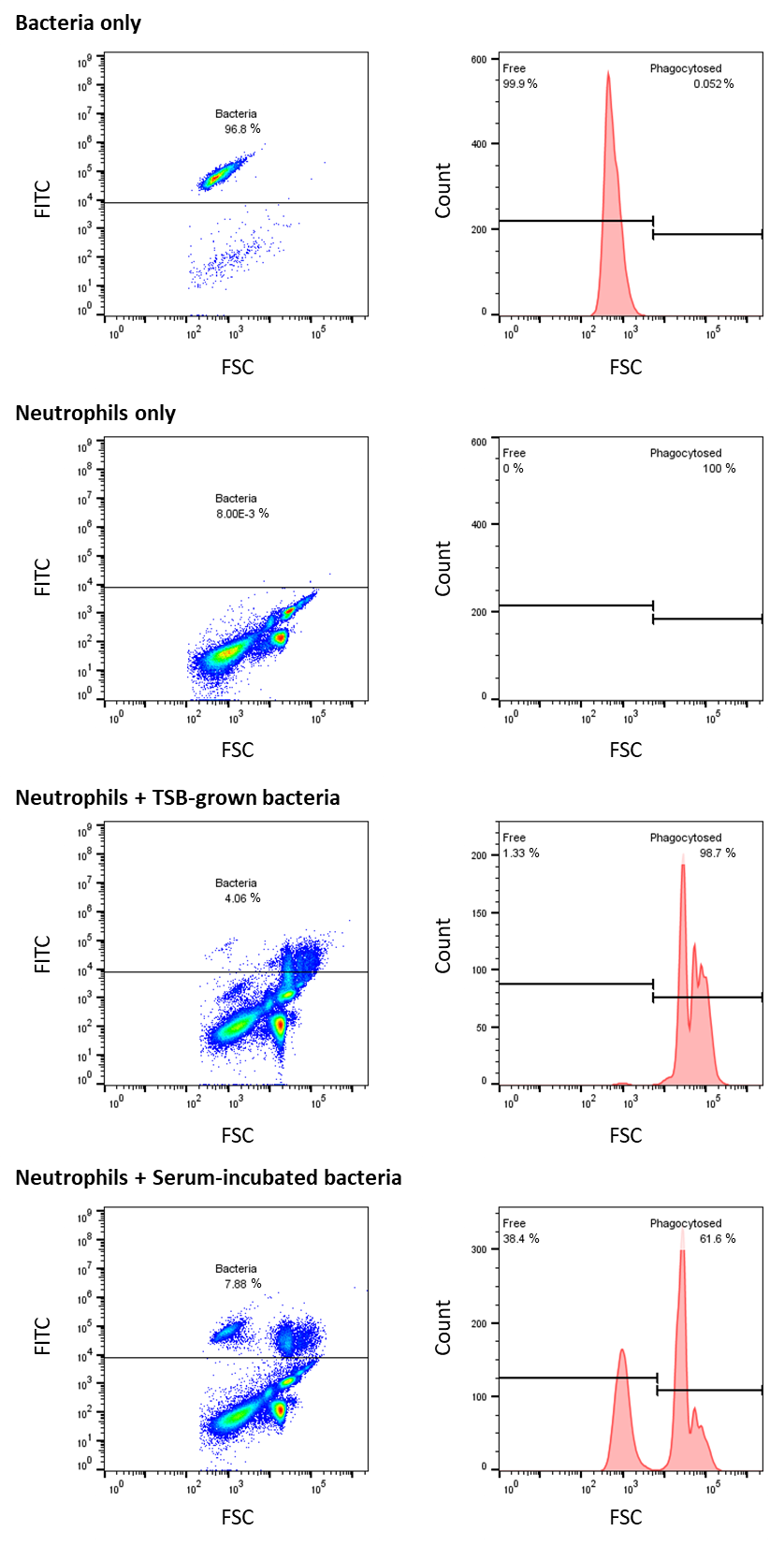


**Supplementary figure S2. Gating strategy for flow cytometry-based opsonophagocytosis assay.** Bacteria were labelled with FITC and gated based on fluorescence (A) and FSC (B). Neutrophils were not fluorescent (C) and had a greater FSC than bacteria (D). This enabled us to detect a shift in FSC of fluorescent bacteria when they were phagocytosed, which happened at high efficiency for broth grown bacteria (E, F). By contrast, many serum-incubated bacteria remained un-phagocytosed by neutrophils (G, H).


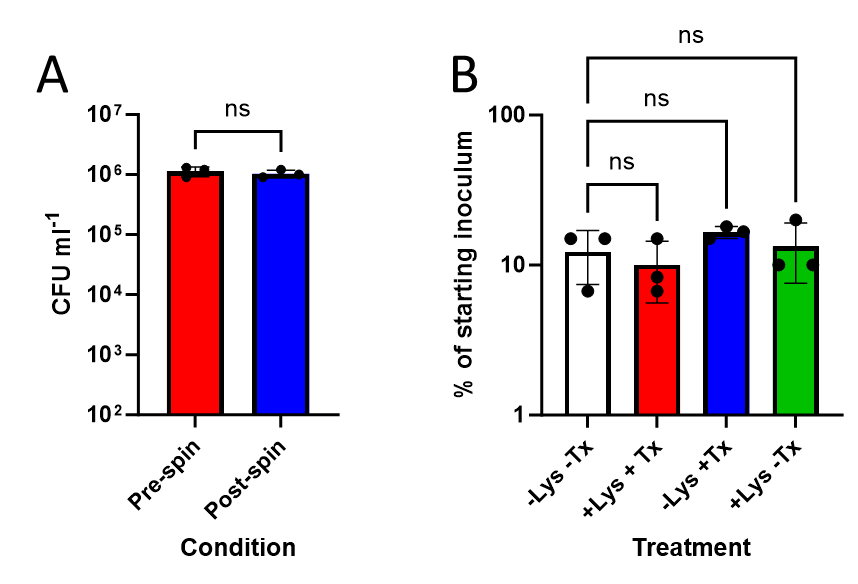


**Supplementary figure S3. Neutrophil-associated bacteria are intracellular.** One of the phagocytosis assays used relies on a centrifugation step (500 x *g*, 1 min) to separate free bacteria from those associated with neutrophils. To demonstrate that this centrifugation step was appropriate, *S. aureus* cells were suspended in HBSS and then centrifuged at 500 x *g* (1 min). CFU counts in the supernatant before and after centrifugation were determined for three independent experiments (**A**). This demonstrated that this centrifugation step does not pull-down free bacteria out of suspension.

Since bacteria that are pulled down via association with neutrophils could be phagocytosed or attached to the outside, we examined the impact of lysostaphin (Lys) which will kill extracellular but not intracellular bacteria [55,56], on CFU counts of bacteria associated with neutrophils (**B**). Since the presence of lysostaphin had no effect on CFU counts of bacteria associated with neutrophils, we can conclude that neutrophil-associated bacteria have been phagocytosed.

Next, we determined whether treatment of neutrophils with Triton X-100 (Tx) increased recovery of phagocytosed bacteria (**B**), since some assays use a detergent to promote lysis of the immune cell, whilst others do not [54,56]. However, the presence of the detergent had no effect on CFU counts of bacteria phagocytosed by neutrophils. As such, we did not employ detergent in subsequent assays.

In (**A**), bars show the mean of 3 independent experiments. Error bars show the standard deviation. Data were analysed by student’s t-test. In (**B**) bars show the mean of 3 independent experiments. Error bars show the standard deviation. Data were analysed by one-way ANOVA and Dunnett’s post hoc test for multiple comparisons (ns p>0.05).

**Supplementary figure S4. Increased evasion of phagocytosis by serum-incubated *S. aureus* relative to TSB-grown bacteria is not dependent on growth phase.** Stationary phase bacteria were incubated in 100% human serum for 16 h, or not, before incubation with neutrophils in 10% human serum for 30 min, before the number of living cells that were not associated with neutrophils (free) (A) or were neutrophil-associated (B) quantified by CFU counts. Similar to exponential phase bacteria, serum-incubated *S. aureus* evaded phagocytosis (free) at significantly higher levels than bacteria that had not been incubated in serum. There was no difference in the number of CFU associated with neutrophils between TSB grown and serum-incubated stationary phase *S. aureus*. Bars show the mean of 4 independent experiments, with each data point indicated with a circle. Data were analysed by paired two-tailed student’s t-test. * p = 0.024, ns not significant (p = ≥ 0.05).


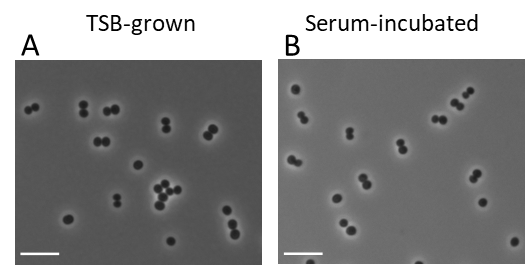


**Supplementary figure S5. Reduced phagocytosis of serum-incubated *S. aureus* is not due to the formation of bacterial aggregates.** Light microscopy images of TSB-grown (A) and serum-incubated (B) *S. aureus* cells shows that there is no difference in aggregation between the two conditions. Scale bars, 5μm.

**Supplementary figure S6. A Δ*spa* *sbi*::Tn double mutant does not bind IgG Fc.** To ensure that the constructed *S. aureus* JE2 Δ*spa* *sbi*::Tn double mutant did not bind IgG, we measured the adhesion of biotinylated IgG Fc fragment to the surface of *S. aureus* JE2 wild type (WT) and mutant strains. As expected, wild type *S. aureus* bound high levels of IgG Fc whilst a *sbi*::Tn mutant showed reduced binding and there was no detectable binding by the Δ*spa* *sbi*::Tn double mutant. Bars show the mean of 4 independent experiments. Error bars show the standard deviation. Data were analysed by one-way ANOVA and Dunnett’s post hoc test for multiple comparisons (**p=<0.01, ***p=<0.001).


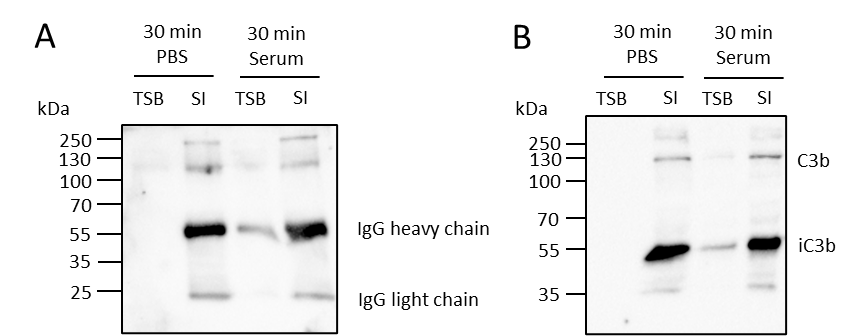


**Supplementary figure S7. Serum-incubated bacteria have large amounts of bound antibody and complement.** TSB-grown and serum-incubated (100%) (SI) *S. aureus* cells were incubated for 30 min with PBS or 10% fresh serum before the levels of bound (**A**) IgG and (**B**) C3 were determined by western blotting. The expected positions for IgG heavy and light chains (**A**) and C3b and ic3b (**B**) are indicated. As reported previously, we observed processing of C3b to iC3b on the *S. aureus* cell surface [59].


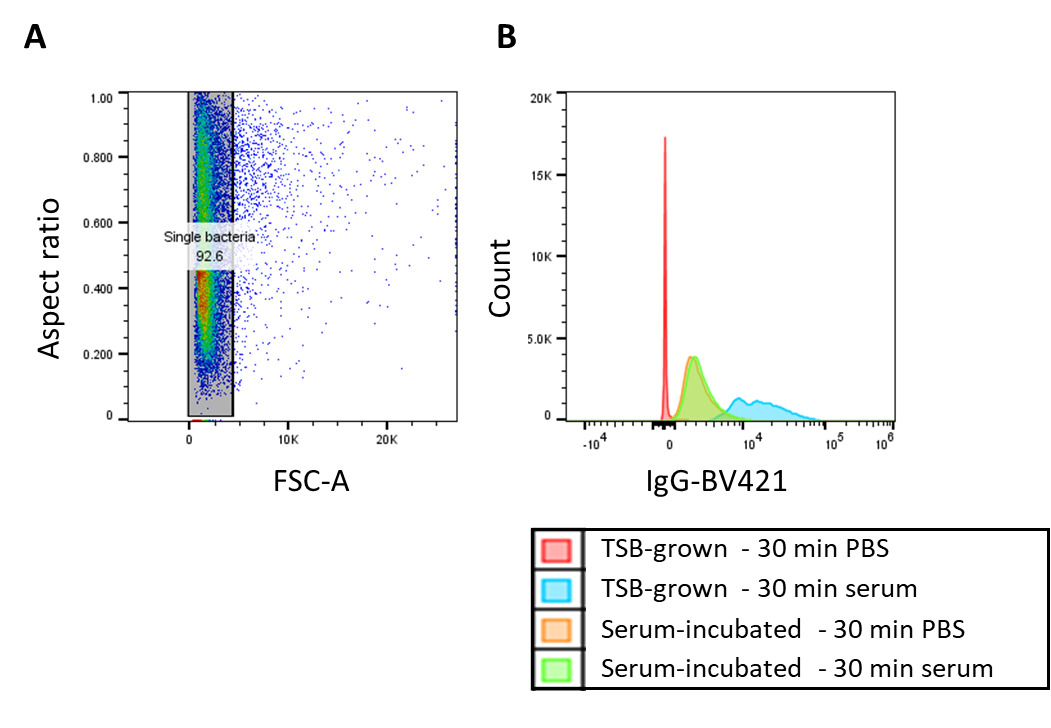


**Supplementary figure S8. Gating strategy for flow cytometry-based determination of opsonin exposure.** Bacteria were grown in broth or incubated in human serum (100%) before labelling with antibodies against complement C3 component or human antibody. Bacteria were gated based on FSC values to select single cells (A). The fluorescence of each single cell was then measured to generate median fluorescence values for each experimental condition. For example, bacteria grown in broth, or incubated in serum and then exposed, or not, to fresh serum (B). This shows that, for broth-grown bacteria that had not been exposed to serum there was no fluorescence, indicative of an absence of non-specific binding. However, for broth grown bacteria exposed to serum there was a strong fluorescence signal (B). Serum-incubated bacteria were moderately fluorescent, regardless of whether they had been exposed to fresh serum or not (B).

**Supplementary figure S9. Antibiotics that inhibit cell wall synthesis promote accessibility of bound IgG against LTA and surface proteins during serum-induced cell wall remodelling.** *S. aureus* was grown in TSB to exponential phase before incubation in serum for 30 min to enable opsonisation to occur (TSB), or for 16 h to allow cell wall remodelling (Ser) +/- the following antibiotics: oxacillin (Oxa), fosfomycin (Fos), AFN-1252 (AFN) or ciprofloxacin (Cip). Subsequently, surface exposed IgG was eluted and then assayed for binding to major surface structures wall teichoic acid (WTA), peptidoglycan (PG), lipoteichoic acid (LTA), membrane-associated proteins (MP) or cell wall-associated proteins (CWP). Data were normalised to values for the TSB condition and represent the median ± 95% CI of three independent biological replicates and were analysed by Kruskal Wallis test and Dunn’s *post-hoc* test to establish statistically significant differences between groups (***, P <0.001; **, P < 0.01; *, P < 0.05; ns, P ≥ 0.05 for the indicated comparisons).

**Supplementary figure S10. Host adaptation in the presence of AFN-1252 does not lead to increased phagocytic killing of *S. aureus*.** Survival of TSB-grown (TSB) and serum-incubated wild type *S. aureus* USA300 (Serum) or incubated in serum containing AFN-1252, which inhibits fatty acid synthesis. Data represent the mean ± standard deviation of three independent biological replicates. Data were analysed by a two-way ANOVA with Sidak’s *post-hoc* test (the presence of AFN-1252 during serum-incubation did not affect susceptibility to neutrophil-mediated killing P > 0.05).


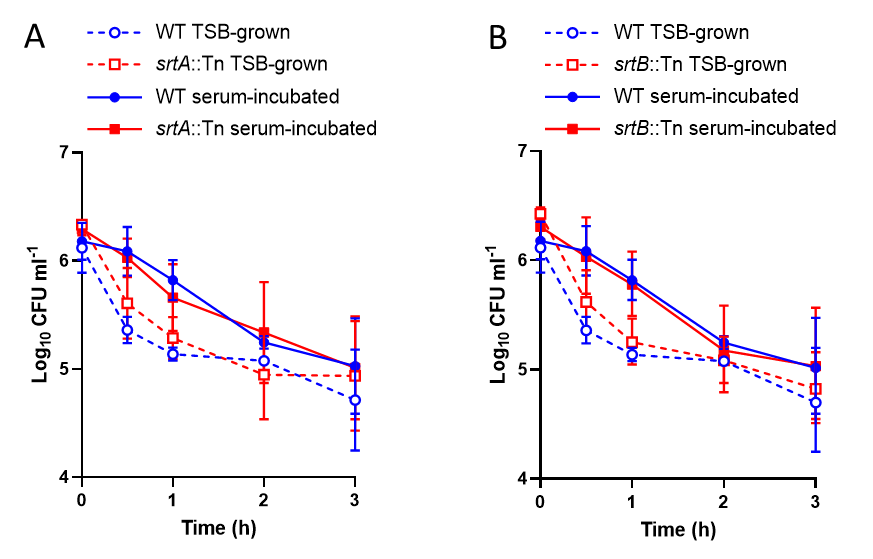


**Supplementary figure S11. Peptidoglycan-anchored proteins do not contribute to immune evasion in serum-incubated *S. aureus*.** Wild type and mutant bacteria were grown in broth or incubated in 100% human serum before incubation with human neutrophils in the presence of serum and staphylococcal survival measured over time. (**A**), Log10 CFU ml^-1^ survival of TSB-grown and serum-incubated cultures of wild type *S. aureus* USA300 or a *srtA*::Tn mutant that lacks peptidoglycan-bound LPXTG-motif proteins. (**B**), Log10 CFU ml^-1^ survival of TSB-grown and serum-incubated cultures of wild type *S. aureus* USA300 or a *srtB*::Tn mutant that lacks peptidoglycan-bound NPQTN-motif protein IsdC. Data were analysed by a two-way ANOVA (neither mutant was significantly affected for survival compared to wild type, P > 0.05). All data were generated in the same experiments, but values for *srtA*::Tn and *srtB*::Tn are shown on separate graphs for clarity. Data for WT cells are the same in both graphs.


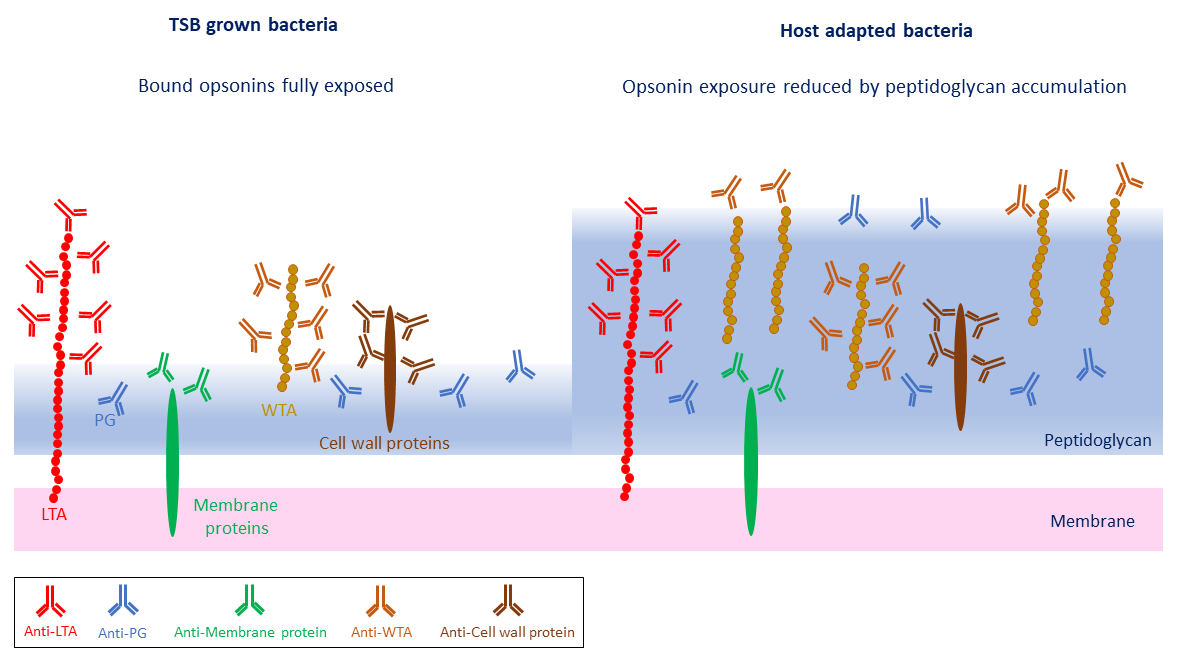


**Supplementary figure S12. Summary diagram of how the adaptation of *S. aureus* to the host environment modulates the exposure of bound opsonins.** TSB grown bacteria have high levels of exposed surface structures and antibodies that target them, resulting in efficient opsonophagocytic killing. However, as *S. aureus* adapts to the host, peptidoglycan accumulates, which conceals many of these bound antibodies, resulting in reduced opsonophagocytic killing.
